# Comparative Subcellular Proteomics Analysis of Susceptible and Near-isogenic Resistant *Bombyx mori* (Lepidoptera) Larval Midgut Response to BmNPV infection

**DOI:** 10.1038/srep45690

**Published:** 2017-03-31

**Authors:** Xue-yang Wang, Hai-zhong Yu, Jia-ping Xu, Shang-zhi Zhang, Dong Yu, Ming-hui Liu, Lin-ling Wang

**Affiliations:** 1 School of Life Sciences, Anhui Agricultural University, Hefei, 230036, People’s Republic of China; 2 Institute of Sericulture, Anhui Academy of Agricultural Sciences, Hefei, 230061, People’s Republic of China; 3 School of Life Sciences, Chongqing Normal University, Hefei, 401331, People’s Republic of China.

## Abstract

The molecular mechanism of silkworm resistance to Bombyx mori nucleopolyhedrovirus (BmNPV) infection remains largely unclear. Accumulating evidence suggests that subcellular fractionation combined with proteomics is an ideal technique to analyse host antiviral mechanisms. To clarify the anti-BmNPV mechanism of the silkworm, the near-isogenic line BC9 (resistant strain) and the recurrent parent P50 (susceptible strain) were used in a comparative subcellular proteomics study. Two-dimensional gel electrophoresis (2-DE) combined with mass spectrometry (MS) was conducted on proteins extracted from the cytosol, mitochondria, and microsomes of BmNPV-infected and control larval midguts. A total of 87 proteins were successfully identified from the three subcellular fractions. These proteins were primarily involved in energy metabolism, protein metabolism, signalling pathways, disease, and transport. In particular, disease-relevant proteins were especially changed in microsomes. After infection with BmNPV, differentially expressed proteins (DEPs) primarily appeared in the cytosolic and microsomal fractions, which indicated that these two fractions might play a more important role in the response to BmNPV infection. After removing genetic background and individual immune stress response proteins, 16 proteins were identified as potentially involved in repressing BmNPV infection. Of these proteins, the differential expression patterns of 8 proteins according to reverse transcription quantitative PCR (RT-qPCR) analyses were consistent with the 2-DE results.

The silkworm *Bombyx mori* L. (Lepidoptera: Bombycidae) has been domesticated for more than 5000 years and still plays an important role in many developing countries. *B. mori* is also a good model organism for the study of insect genetics and immunology^1^,^2^. Bombyx mori nucleopolyhedrovirus (BmNPV) is a primary silkworm pathogen and annually causes serious economic losses. Notably, some silkworm strains exhibit high resistance to BmNPV infection^3^, but the molecular mechanisms of the resistance to BmNPV infection have not been fully elucidated.

In recent decades, a series of studies of the silkworm response to BmNPV infection have been reported. *B. mori* serine lipase-1, protease-2, and alkaline trypsin protein extracted from the digestive juice of the larval midgut have been reported to exhibit strong antiviral activity *in vitro*^4^,^5^,^6^. NADH-oxidoreductase-like protein inhibited the capability of BmNPV particles to infect BmN cells *in vitro*^7^. Arginine kinase was found to be involved in silkworm resistance to BmNPV infection using 2-DE to analyse the proteomes of different resistant strains^8^. Beta-N-acetylglucosaminidase 2 and aminoacylase were shown to be up-regulated in BmNPV-resistant strains using 2-DE to compare differences in protein patterns in different resistant strains hemolymph^9^. In our laboratory, a total of 12 proteins potentially related to viral infection were obtained using one- and two-dimensional electrophoresis, followed by virus overlay assays. The functions of these proteins can be classified into three groups: endocytosis, intracellular transportation, and host responses^10^. However, these previous results do not clarify the molecular mechanism of silkworm resistance to BmNPV.

Recently, comparative subcellular proteomics has become a useful strategy to reduce sample complexity and protein overlapping in exploring disease-resistant mechanisms^11^,^12^. As “energy factories,” mitochondria are related to the chemical energy metabolism of adenosine triphosphate (ATP) and thus are involved in cell cycle and growth, as well as other biological processes, such as signal transduction, cellular differentiation, and cell death^13^. In addition to their well-appreciated roles in biological processes, mitochondria appear to function as centrally positioned hubs in viral infection; some viruses have been reported to regulate host cell apoptosis based on mitochondrial regulation, including human immunodeficiency virus (HIV), influenza A virus (IAV), and hepatitis C virus (HCV)^14^. The microsome is a vesicle-like structure that is re-formed from pieces of endoplasmic reticulum and primarily participates in endoplasmic reticulum-like protein synthesis, protein glycosylation, and lipid synthesis. The differential expression of microsomal proteomes is consistently related to extraneous pathogen infection. Microsomes from DCs armed with peptide-MHC complexes can be an important alternative to DC-based vaccines for protection against viral infection^15^. The cytosol contains multiple levels of organization, including concentration gradients of various ions, large complexes of enzymes, the cytoskeleton, and protein compartments^16^,^17^,^18^. Almost all biological reactions must rely on certain cytosolic components or occur in the cytosol. Thus, understanding the cytosolic proteome is also crucial. Subcellular proteomics can largely reduce the complexity of protein samples and spot overlapping. Furthermore, to our knowledge, differentially expressed proteins (DEPs) in different resistant silkworm strains following BmNPV infection have not been analysed based on subcellular proteomics.

In this study, subcellular proteomic maps of the near-isogenic line BC9 (resistant strain) and the recurrent parent P50 (susceptible strain) midguts following BmNPV infection were constructed and analysed based on three subcellular protein fractions: cytosol, mitochondria, and microsome. Many DEPs were identified in the three subcellular fractions using 2-DE combined with matrix-assisted laser desorption/ionization-time-of-flight mass spectrometry (MALDI-TOF MS); some of these proteins were potentially involved in resisting BmNPV infection. Our study provides an overview of the subcellular proteomic profiles of silkworm responses to BmNPV infection and lays a foundation for clarifying the mechanism of silkworm resistance to BmNPV.

## Materials and Methods

### Silkworm and virus

The recurrent parent P50 (susceptible strain) and donor parent A35 (resistant strain) were maintained in the Key Laboratory of Sericulture, Anhui Agricultural University, Hefei, China. The near-isogenic line BC9 was constructed according to the protocol of Yao *et al*.^19^. In brief, the recurrent parents were crossed with the donor parents; progeny were repeatedly backcrossed with the recurrent parents for nine generations, and each progeny was screened with BmNPV. Thus, the genetic background of BC9 is very similar to the recurrent parent P50, but BC9 should have the resistant background derived from A35.

BmNPV (T3 strain) was maintained in our laboratory and purified according to the protocol reported by Rahman *et al*.^20^. A haemocytometer was used to calculate the concentration of BmNPV (OB/mL).

### Silkworm resistance level bioassays

The level of silkworm resistance to BmNPV was assayed according to the protocol of Cheng *et al*.^21^. In brief, the first day of fourth instar larvae were infected with different concentrations of BmNPV; inoculations were conducted in triplicate. The resistance level of the silkworm was calculated using IBM SPSS Statistics 20 (IBM,USA).

### Silkworm midgut sample preparation

On the first day of fifth instar, all larvae were starved for 24 h. Then, 30 silkworms from each group were treated orally with 5 μL of BmNPV suspended in sterile water (1.0 × 10^5^ OB/mL) per larva. The rapid proliferation of BmNPV was detected at approximately 24 hours post-inoculation (hpi)^22^; thus, this time was considered optimal for sample collection. Silkworm larvae were dissected, and midgut tissues were removed and then washed in DEPC (Sangon, China)-treated H_2_O. Thirty larval midguts were mixed together to minimize individual genetic differences. Samples were flash-frozen in liquid nitrogen and pulverized, and 100 mg of each sample was placed directly into RNase-free microcentrifuge tubes containing 1.0 mL of TRIzol Reagent (Invitrogen, USA) and stored at −80 °C for later use.

### Subcellular protein extraction

Subcellular proteins were extracted according to the method described by Lu *et al*. with some modification^23^,^24^. A total of 50 mg of pulverized midgut sample was homogenized in 500 μL of potassium phosphate buffer (pH 7.6) containing 0.1 M potassium phosphate and 1.0 mM PMSF. The homogenate was centrifuged at 1,000 × *g* for 5 min at 4 °C to remove debris. The supernatant (total protein) was transferred to a new tube for the next step. To fractionate mitochondrial, microsomal and cytosolic proteins, the supernatant was further centrifuged at 10,000 × *g* for 10 min at 4 °C. The pellet was collected as mitochondrial proteins and then washed three times with potassium phosphate buffer, centrifuged at 10,000 × *g* for 10 min at 4 °C, and re-suspended in deionized water. The supernatant containing cytosolic and microsomal proteins was transferred to a new tube, mixed with an equal volume of 16 mM CaCl_2_, incubated on ice for 5 min, and then centrifuged at 10,000 × *g* for 10 min at 4 °C. The pellet was collected as microsomal proteins and washed three times with potassium phosphate buffer, centrifuged at 10,000 × *g* for 10 min at 4 °C, and then re-suspended in deionized water. The resulting supernatant (cytosolic protein source), mitochondrial proteins, and microsomal proteins were transferred into 4 × volumes of precooled acetone, and incubated overnight at −20 °C. The fractions were then centrifuged at 10,000 × *g* for 10 min at 4 °C and washed three times with precooled acetone. Only the cytosolic proteins were cleaned using the 2-D Clean-up Kit (GE Healthcare, USA) according to the manufacturer’s instructions. The final proteins in the three subcellular fractions were dissolved in a rehydration solution containing 7 M urea, 2 M thiourea, 60 mM DTT, 65 mM CHAPS, 2% Triton X-100, and 0.2% ampholytes 5–8 for later use. The extraction of subcellular protein was performed in triplicate. The protein concentration was calculated using the Bradford method^25^.

### Sodium dodecyl sulfate polyacrylamide gel electrophoresis (SDS-PAGE)

For the SDS-PAGE procedure, 5× loading buffer (50 mM Tris-HCl, pH 8.0, 250 mM DTT, 5% SDS, 50% glycerol, and 0.04% bromophenol blue) was added to the protein sample. Samples were boiled for 8 min and subjected to SDS-PAGE with a 12% SDS polyacrylamide gel. Electrophoresis was performed on the Mini-protean Tetra system (Bio-Rad, USA), and gels were stained with Coomassie brilliant blue R250.

### Two-dimensional gel electrophoresis (2-DE)

For 2-DE, 800 μg of each sample protein dissolved in 300 μL of rehydration solution with 0.3 μL of 1% bromophenol blue dye was loaded onto a 17-cm immobilized linear dry strip (pH 5–8, Bio-Rad, USA). The strip was actively rehydrated at 20 °C for 13 h at 50 V. The rehydrated strip was automatically focused using the following program: 100 V, slow, 1 h; 200 V, slow, 1 h; 300 V, slow, 1 h; 500 V, slow, 2 h; 1,000 V, slow, 2 h; 5,000 V, slow, 2 h; 10,000 V, linear, 2 h; 10,000 V, rapid, 80,000 V.h; and 500 V, rapid, 24 h^26^. The current for each strip was limited to 50 μA. After IEF separation, the strips were immediately equilibrated with gentle shaking for 14 min 30 s in equilibration buffer (6 M urea, 20% glycerol, 2% SDS and 0.375 mM Tris-HCl, pH 8.8) containing 2% (w/v) DTT, followed by an equilibration for 14 min 30 s in the above equilibration buffer but containing 2.5% (w/v) IAM instead of DTT. Equilibrated IPG strips were further separated on a 10% SDS-PAGE gel. The procedures were performed at 10 mA/gel for 30 min and then 30 mA/gel until the bromophenol blue dye ran out from the bottom of the gels.

### Gel visualization and image analysis

For visualization, gels were stained with Coomassie brilliant blue G-250. The stained gels were scanned at a resolution of 600 dpi. Spot detection, matching and quantitative intensity analysis were performed using PDQuest software (version 8.0, Bio-Rad, USA). Each protein sample was analysed in triplicate. Differential analyses of images were performed by matching the spots of the control and experimental groups. The unique protein spots or significantly differentially expressed protein spots (more than 2-fold) were selected and subjected to MS identification.

### MALDI-TOF MS and database searching

In-gel digestion was performed as described earlier^27^. Differentially expressed protein spots were excised from the stained gels, washed twice with Milli-Q water, and de-stained at room temperature for 5 min. The de-staining solution was removed, and samples were washed twice and incubated in 50% acetonitrile for 5 min, followed by removal of the acetonitrile and addition of 100% acetonitrile for 5 min. The gel was rehydrated in 4.0 μL of trypsin solution (Promega, Madison, USA) (20 μg/mL in 25 mM NH_4_HCO_3_) for 30 min. Next, 20 μL of cover solution (25 mM NH_4_HCO_3_) was added for digestion at 37 °C for 16 h. The supernatant was transferred into a new tube and extracted once with 50 μL of extraction buffer (67% acetonitrile and 5% TFA). The peptide extract and supernatant were combined and then completely dried. The prepared sample was re-suspended with 5.0 μL of 0.1% TFA, followed by mixing with an equal volume of a matrix consisting of a saturated solution of α-cyano-4-hydroxy-trans-cinnamic acid in 50% acetonitrile and 0.1% TFA; 1.0 μL of mixture was spotted onto a new target plate. Peptide MS and MS/MS data were obtained with an ABI 5800 MALDI-TOF/TOF Plus mass spectrometer (Applied Biosystems, USA). The data were obtained in a positive MS reflector using a CalMix5 standard to adjust the instrument (ABI5800 Calibration Mixture). The GPS Explorer V3.6 software (Applied Biosystems, USA) with default parameters was used to integrate and process both the MS and MS/MS data.

Database searching was performed based on a 95% or higher confidence interval of the scores of proteins in the Mascot V2.3 search engine (Matrix Science Ltd., U.K.) using the following parameters: NCBInr database; trypsin as the digestion enzyme; one missed cleavage site; fixed modifications of carbamidomethyl (C); partial modifications of acetyl (Protein N-term), deamidated (NQ), dioxidation (W), oxidation (M); 100 ppm for precursor ion tolerance and 0.5 Da for fragment ion tolerance.

### Protein identification and annotation

Protein identification used the following criteria: peptide mass fingerprinting was considered sufficient for identification if several peptides specific for a given protein were found, and protein identifications were accepted if they could be determined with greater than 95.0% probability and contained at least two unique peptides. Additionally, the calculated molecular weight (MW) and isoelectric point (p*I*) resulting from the MASCOT analysis agreed with the observed values to the greatest extent possible. Significant annotation was also associated with the identified organism and function in relevant biological contexts. Identified proteins were annotated based on the literature and information available in various databases, including the NCBI, Swiss-Prot/TrEMBL, and Gene Ontology (GO) databases.

### Bioinformatics analysis

In living cells, many proteins can interact with each other, and these interacting proteins are expected to be involved in the same biological process or to handle in the same subcellular compartment, which is supported by the evidence that proteins in the same pathway are more interconnected^28^. STRING (http://string-db.org/) contains abundant resources on physical and functional interactions and collects information from numerous sources, including experimental repositories, computational prediction methods, and public text collections^29^. In this study, STRING was adopted to analyse the protein-protein interactions (PPIs) of the selected proteins. Due to the lack of proteomics information on *B. mori* in STRING, the PPIs network was built using the database of another well-studied insect, *Drosophila melanogaster (D. melanogaster*).

The related KEGG pathways of the selected proteins were analysed according to the method used by Qin *et al*.^30^. Briefly, the annotations of identified proteins were obtained by searching UniprotKB (http://www.uniprot.org/) using *Bombyx mori* and *Drosophila* database; then, KEGG pathways were analysed in accordance with the KEGG database (http://www.genome.ad.jp/kegg/pathway.html).

### RNA isolation and cDNA synthesis

Total RNA of silkworm midgut was extracted with TRIzol Reagent (Invitrogen, USA) according to the manufacturer’s instructions. A NanoDrop 2000 spectrophotometer (Thermo Scientific, USA) was used to quantify the concentrations. The purity of all RNA samples were assessed at absorbance ratios of A_260/280_ and A_260/230_, and the integrity of the RNA was confirmed by 1% agarose gel electrophoresis. The first strand cDNA was synthesized using an RT reagent kit (TaKaRa, Japan) according to the manufacturer’s instructions.

### Reverse transcription Quantitative PCR (RT-qPCR)

To confirm the results of selected proteins, the relative expression levels of these proteins were validated by RT-qPCR assays. Primers used in the RT-qPCR assays are shown in Supplementary Table S1. RT-qPCR reactions were prepared with a SYBR Premix Ex Taq^TM^ Kit (TaKaRa, Japan) according to the manufacturer’s instructions. The reactions were carried out in the CFX96TM Real-Time System (Bio-Rad, USA). The thermal cycling profile consisted of an initial denaturation at 95 °C for 30 s and 40 cycles at 95 °C for 5 s and 60 °C for 30 s. All assays were performed in triplicate. Relative expression levels were calculated using the 2^−ΔΔCt^ method. In this study, *B. mori* glyceraldehyde-3-phosphate dehydrogenase (*BmGAPDH*) was selected as an internal control. Statistical analyses were conducted using the SPSS software (IBM, USA).

## Results

### Resistance to BmNPV of different silkworm strains

The median lethal concentration (LC_50_) was used to evaluate the level of silkworm resistance to BmNPV. In this study, the LC_50_ value of A35 was approximately 26-fold greater than that of BC9 and over 500-fold greater than that of P50. The value of BC9 was 23-fold greater than that of P50 (Table 1).

### Variations in protein banding patterns among the subcellular protein fractions

**Figure 1:**
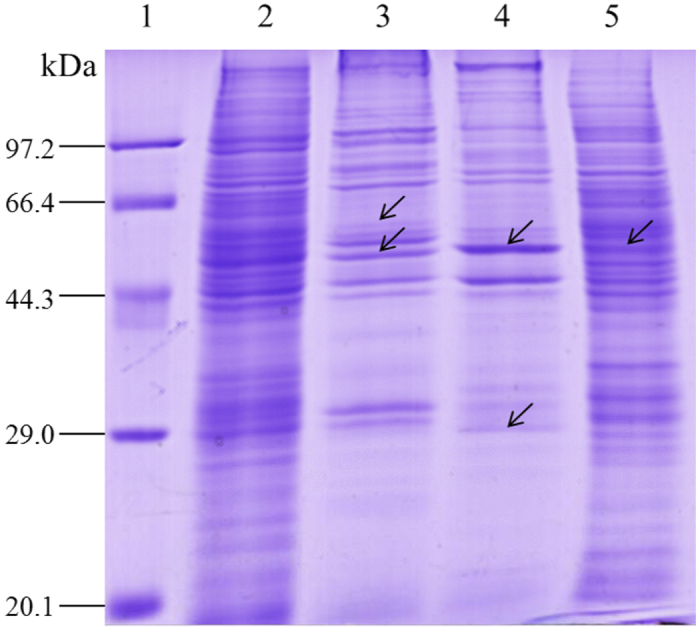
Analysis of subcellular protein fractions of P50 midgut by SDS-PAGE. 1, markers; 2, total proteins; 3, mitochondrial proteins; 4, microsomal proteins; 5, cytosolic proteins. A quantity of 35 μg of each protein sample was loaded and electrophoresed in a 12% polyacrylamide gel and stained with Coomassie brilliant blue R250. Arrows represented differential and the same bands in the subcellular fractions. The full-length gels were included in the Supplementary Fig. S1.

It is essential to note that complete purification of subcellular fractions is nearly impossible, with few exceptions. However, subcellular fractionation is still a flexible and adjustable approach to reducing sample complexity and protein overlapping^31^. In this study, total protein and three subcellular protein fractions (mitochondrial, microsomal, and cytosolic) of the silkworm midgut were extracted. To detect the quality of the subcellular proteins, SDS-PAGE was used. Many bands with varying levels of intensity were observed among the three subcellular fractions. The bands in the total protein sample had matching bands with the same MW in one or more of the subcellular protein fractions; for example, 55 kDa bands existed in all of the subcellular fractions, whereas the 57 and 29 kDa bands had only one matching band in the mitochondrial and microsomal fractions, respectively. Notably, some matching bands in the subcellular protein fractions exhibited much higher intensity compared with the total protein (Fig. 1). Overall, the subcellular protein samples were suitable for subsequent 2-DE analysis.

### Changes in the cytosolic proteome profile in different resistant strains following BmNPV infection

**Figure 2:**
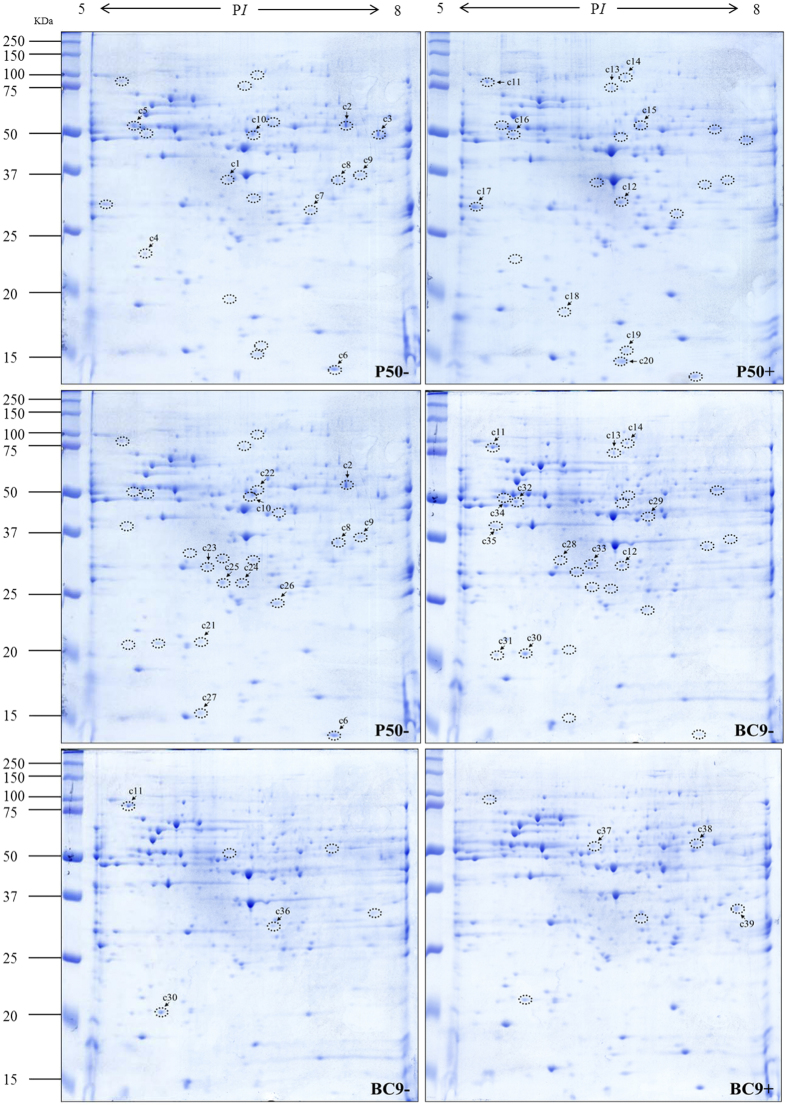
2-DE images of cytosolic protein extracts from P50 and BC9 following BmNPV infection. P50−, treated with sterile water; P50+, infected with BmNPV; BC9−, treated with sterile water; BC9+, infected with BmNPV. Proteins were separated by pH 5–8 IPG strips, followed by SDS-PAGE on 10% gels. The gels were stained with Coomassie brilliant blue G-250. DEPs are marked by a label with a number. All samples were processed in parallel. The full-length gels were included in the Supplementary Fig. S2.

Initially, IPG strips that ranged from pH 3 to 10 were used to analyse DEPs in the two strains following BmNPV infection. However, the dense area of spots was primarily distributed in the regions from pH 5 to 8; therefore, pH 5–8 IPG strips were used to improve spot resolution. Three repetitions for each sample were analysed using PDQuest software. Based on the normalized volume of each spot, DEPs were analysed in two strains following BmNPV infection. A large number of DEPs were obtained from the comparative analysis, but those with a ratio ≥2 were selected for further analysis. Thus, a total of 38 spots were determined to be differentially expressed in the cytosol (Fig. 2), and the results are summarized in Table 2. The number of DEPs in P50+ vs. P50−, BC9− vs. P50−, and BC9+ vs. BC9− was 20, 14, and 4, respectively. In P50+ vs. P50−, a half number of proteins were up-regulated. In BC9− vs. P50−, 8 proteins were up-regulated, whereas in BC9+ vs. BC9−, 3 proteins were down-regulated (Table 2).

### Identification of differentially expressed mitochondrial proteins in different resistant strains following BmNPV infection

**Figure 3:**
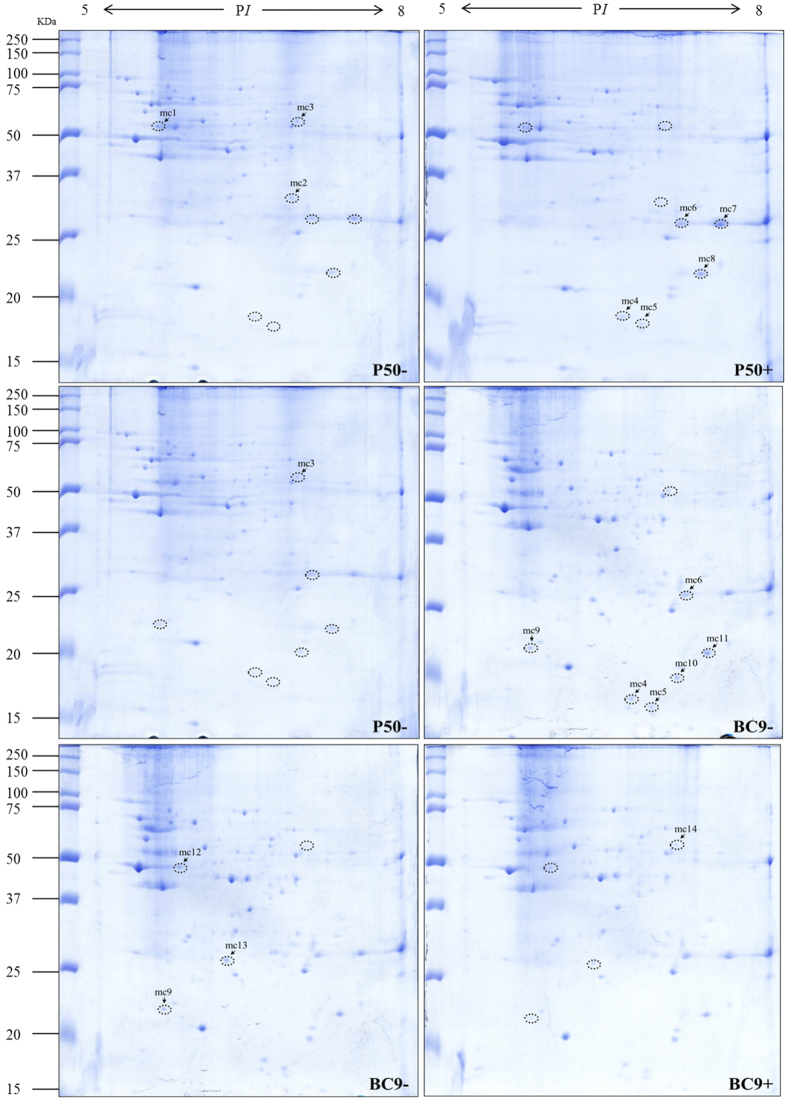
2-DE images of mitochondrial protein extracts from P50 and BC9 following BmNPV infection. P50−, treated with sterile water; P50+, infected with BmNPV; BC9−, treated with sterile water; BC9+, infected with BmNPV. Proteins were separated with pH 5–8 IPG strips, followed by SDS-PAGE on 10% gels. The gels were stained with Coomassie brilliant blue G-250. DEPs are marked by a label with a number. All samples were processed in parallel. The full-length gels were included in the Supplementary Fig. S3.

Fractions of mitochondrial proteins from P50 and BC9 following BmNPV infection were also analysed using pH 5–8 IPG strips. The 2-DE images of mitochondrial proteins are shown in Fig. 3. The DEPs were filtered according to the method described above. After removing unqualified spots, 14 DEPs with significant changes were selected for further analysis, and the results are summarized in Table 3. The number of proteins differentially expressed in P50+ vs. P50−, BC9− vs. P50−, and BC9+ vs. BC9− was 8, 3, and 3, respectively. In P50+ vs. P50−, 5 proteins were up-regulated, whereas in BC9− vs. P50−, all the proteins were up-regulated. In BC9+ vs. BC9−, 2 proteins were down-regulated.

### Changes in the microsomal proteome profile in different resistant strains following BmNPV infection

**Figure 4:**
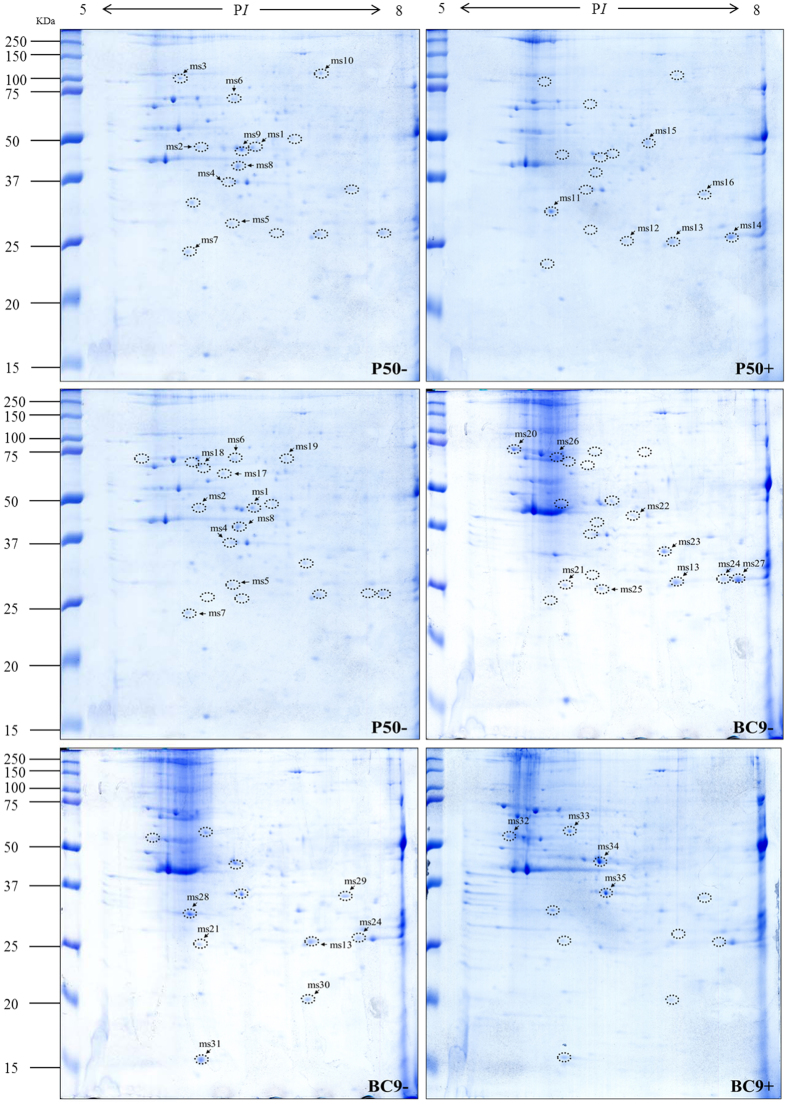
2-DE images of microsomal protein extracts from P50 and BC9 following BmNPV infection. P50−, treated with sterile water; P50+, infected with BmNPV; BC9−, treated with sterile water; BC9+, infected with BmNPV. Proteins were separated with pH 5–8 IPG strips, followed by SDS-PAGE on 10% gels. The gels were stained with Coomassie brilliant blue G-250. DEPs are marked by a label with a number. All samples were processed in parallel. The full-length gels were included in the Supplementary Fig. S4.

Microsomal proteins of the two strains following BmNPV infection were also analysed using pH 5–8 IPG strips. The results for the analysis of microsomal proteins are shown in Fig. 4. After discarding unqualified spots, 35 protein spots were observed to be significantly differentially expressed, and the results are summarized in Table 4. The number of DEPs in P50+ vs. P50−, BC9− vs. P50−, and BC9+ vs. BC9− was 16, 11, and 8, respectively. In P50+ vs. P50−, 10 proteins were up-regulated, and in BC9− vs. P50−, 8 proteins were up-regulated. In BC9+ vs. BC9−, 4 proteins were down-regulated.

### KEGG pathway analysis of differentially expressed proteins (DEPs) in each subcellular fraction

**Figure 5:**
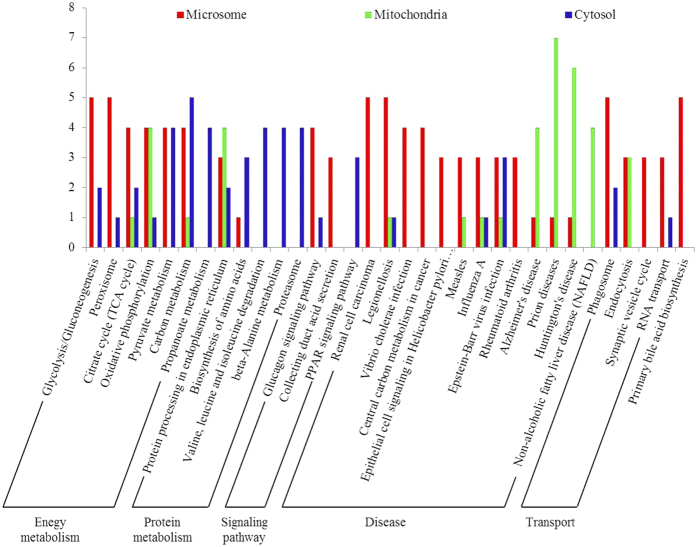
KEGG pathway classification analysis of the DEPs in each subcellular fractions. Numbers represent the total number of DEPs involved in the same pathway.

Among the 87 identified DEPs, 63 proteins were involved in specific KEGG pathways (Fig. 5). To obtain overview information on these proteins, each of the subcellular fractions were individually classified. The relevant pathways were classified into five main categories and 33 subcategories according to the KEGG classifications. In microsomes, proteins that were differentially expressed following BmNPV infection primarily participated in pathways related to energy metabolism (28%), disease (39%), and transport (15%). In the mitochondria, proteins that were differentially expressed mainly participated in energy metabolism (16%) and disease (66%). In the cytosol, proteins that were differentially expressed were primarily involved in energy metabolism (40%) and protein metabolism (35%).

Additionally, the number of up-regulated proteins related to energy metabolism was increased in P50 following BmNPV infection, but decreased in BC9 following virus infection. In relation to protein metabolism, the number of up-regulated proteins was decreased in P50 and increased in BC9 following BmNPV infection. All the proteins were up-regulated in the signalling pathways in P50 following virus infection, whereas only half of the proteins were up-regulated in BC9 following infection. In relation to transport, all the proteins were down-regulated in P50 following virus infection, whereas several proteins were up-regulated in BC9 following infection. After BmNPV infection, the proteins related to primary bile acid biosynthesis were all down-regulated in P50. Notably, we found that a large number of proteins related to disease were altered following BmNPV infection; far more of these proteins were observed in P50 than in BC9, and many were up-regulated in P50 following BmNPV infection (see Supplementary Table S2).

### Analysis of differentially expressed proteins (DEPs) that potentially contribute to BmNPV infection

**Figure 6:**
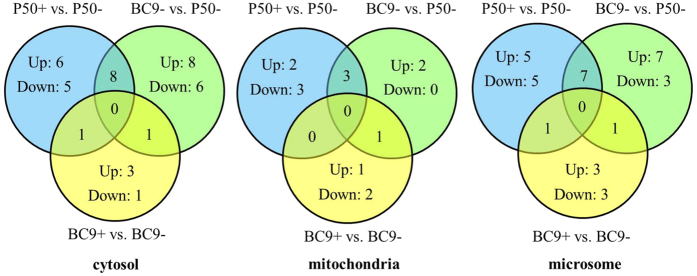
Venn diagram analysis of DEPs in three subcellular fractions of different resistant strains following BmNPV infection.

Those proteins specifically expressed in the susceptible strain were beneficial for analysing the mechanism of silkworm resistance to BmNPV. In this study, 26 proteins (region of blue colour) were uniquely differentially expressed in P50 following BmNPV infection based on a Venn diagram analysis (Fig. 6). There were 11, 5 and 10 proteins distributed in the cytosol, mitochondria, and microsomes, respectively. In the cytosol, 6 proteins were up-regulated in P50 following infection. In the mitochondria, 2 proteins were up-regulated in P50 following infection. In the microsomes, half of the proteins were up-regulated in P50 following infection. Theoretically, these up-regulated proteins may be involved in the BmNPV-stimulated or pathologic response in host cells.

### Analysis of differentially expressed proteins (DEPs) potentially participating in the resistance to BmNPV infection

The near-isogenic line BC9 was constructed based on the recurrent parent P50, and the two strains have a highly similar genetic background. After removing DEPs relevant to the genetic background and immune stress response, 13 proteins (region of yellow colour) that were uniquely differentially expressed in BC9 following infection were obtained according to the Venn diagram analysis (Fig. 6), which were potentially involved in the BmNPV-stimulated or pathologic response. However, the number of varied proteins was significantly lower than that of P50. The number of the DEPs in the cytosol, mitochondria, and microsomes was 4, 3, and 6, respectively. In the cytosol, 3 proteins were up-regulated, and one was down-regulated following infection. In the mitochondria, two proteins were down-regulated, and one was up-regulated following infection. In microsomes, half the number of the proteins was up-regulated following infection.

In particular, certain proteins (overlap region of yellow and green colour) exhibited varied expression levels between the near-isogenic line BC9 (resistant strain) and the recurrent parent P50 (susceptible strain) and were changed in BC9 following infection. Among these proteins, PEBP (c30), NADH dehydrogenase (ubiquinone) Fe-S protein 8 (Ndufs8, mc9), and tudor staphylococcus/micrococcal nuclease (Tudor-SN, ms21) were identified by MS analysis, and these proteins were in the cytosolic, mitochondrial, and microsomal fractions, respectively (Tables 2, 3 and 4). Theoretically, these three proteins may be involved in the response of the resistant silkworm strain to BmNPV infection.

### Protein-protein interactions (PPIs) network analysis of anti-BmNPV-relevant differentially expressed proteins (DEPs)

**Figure 7:**
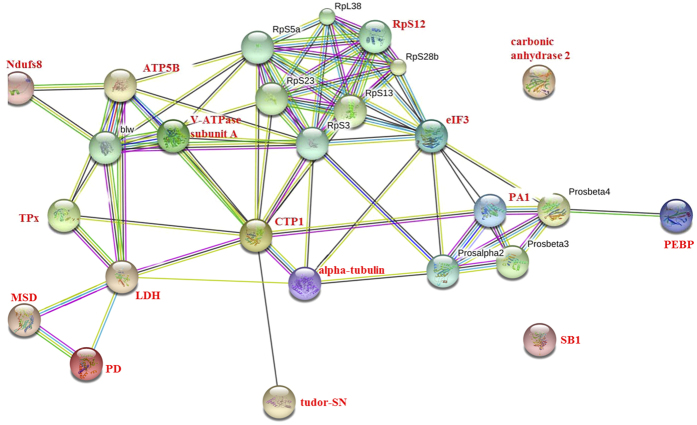
The interaction network of 16 DEPs of interest was constructed based on the STRING website information using the database of another well-studied insect, *D. melanogaster*. The 16 DEPs were highlighted with red font. Tudor-SN, tudor staphylococcus/micrococcal nuclease; V-ATPase subunit A, vacuolar ATP synthase catalytic subunit A; RpS12, ribosomal protein S12; PD, pyruvate dehydrogenase; TPx, thiol peroxiredoxin; eIF3, eukaryotic translation initiation factor 3 subunit I; Ndufs8, NADH dehydrogenase (ubiquinone) Fe-S protein 8; ATP5B, H^+^ transporting ATP synthase beta subunit isoform 2; CTP1, chaperonin containing t-complex polypeptide 1 beta; PEBP, phosphatidylethanolamine binding protein isoform 2; PA1, proteasome subunit alpha type-1 isoform X2; SB1, selenium-binding protein 1 isoform X2; MSD, methylmalonate-semialdehyde dehydrogenase; LDH, L-lactate dehydrogenase.

To further investigate the relationship of the 16 DEPs of interest, which included the 13 proteins (region of yellow colour) uniquely differentially expressed in BC9+ vs. BC9− and the three proteins (overlap region of yellow and green colour) that exhibited varied expression in BC9− vs. P50 and BC9+ vs. BC9−, the functional association of these proteins was analysed using STRING 9.1 online software. A combined score was assigned for every protein-protein association pair in the software. This score was computed by combining the probabilities from several pieces of evidence and correcting for the probability of randomly observing an interaction. As illustrated in Fig. 7, most proteins could be constructed into one network with medium confidence (0.707), except for carbonic anhydrase 2 and selenium-binding protein 1 isoform X2 (SB1). Notably, the multifunctional chaperonin CTP1 had close interaction with its member alpha-tubulin, Tudor-SN, and other functional partners, such as the vacuolar ATP synthase catalytic subunit A (V-ATPase subunit A), H^+^-transporting ATP synthase beta subunit isoform 2 (ATP5B), and Ndufs8, ribosomal protein S12 (RpS12), eukaryotic translation initiation factor 3 subunit I (eIF3), proteasome subunit alpha type-1 isoform X2 (PA1), and PEBP. The chaperonin also closely interacted with energy metabolism-relevant proteins, including L-lactate dehydrogenase (LDH), methylmalonate-semialdehyde dehydrogenase (MSD), pyruvate dehydrogenase (PD), and thiol peroxiredoxin (TPx), and these proteins also can interact closely with each other.

### Validation of anti-BmNPV-relevant differentially expressed proteins (DEPs) using RT-qPCR

**Figure 8:**
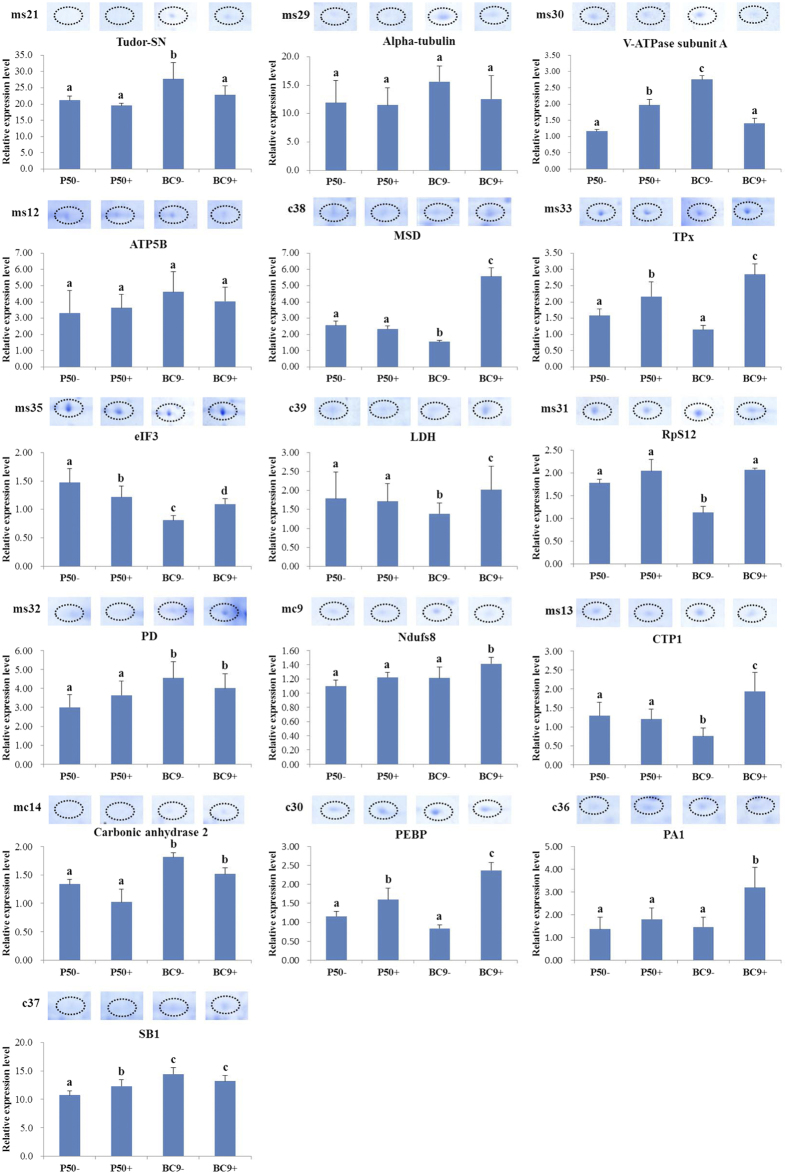
Enlarged spot images and RT-qPCR analysis of the expression levels of anti-BmNPV-relevant DEPs. The data were normalized using *BmGAPDH* and are represented as the means ± standard errors of the means from three independent experiments. Relative expression levels were calculated using the 2^−ΔΔCt^ method. Statistical analysis was conducted using the SPSS software. Significant differences are indicated by different letter, e.g. a, b, c (P < 0.05).

To analyse the differential expression patterns of the 16 DEPs of interest following BmNPV infection, RT-qPCR was used. As illustrated in Fig. 8, 8 proteins exhibited highly similar differential expression patterns at the translational and transcriptional levels in BC9 following infection, including Tudor-SN, alpha-tubulin, V-ATPase subunit A, TPx, eIF3, ATP5B, MSD, and LDH. Additionally, nearly all of these proteins exhibited significant differences in expression in BC9 following infection. However, other proteins displayed different patterns at the translational and transcriptional levels in BC9 following infection. This phenomenon was not unexpected because numerous factors are involved in gene expression, including mRNA stability, splicing, translational regulation, protein post-translational processing, and protein turnover^32^. Nonetheless, proteomics data are more relevant to biological responses because proteins, not RNAs, are the functional products of genes.

## Discussion

Many technologies, such as differential gel electrophoresis and shotgun proteomics, have been used to study the molecular mechanism of silkworm resistance to BmNPV. However, these strategies by nature do not provide a global proteomic profile of silkworm anti-BmNPV mechanisms. Recently, subcellular proteomics combined with MS has been widely adopted in researching disease-resistant mechanisms to reduce the sample complexity and protein coverage, as well as to provide a spatial description of proteins in the cell^31^. In this study, a dynamic overview of the changed host proteins in response to BmNPV infection was obtained by comparing differences in the abundance of proteins isolated from the cytosol, mitochondria, and microsome of the recurrent parent P50 (susceptible strain) and the near-isogenic line BC9 (resistant strain) following BmNPV infection. Many distinctly different protein patterns were observed in 1-DE gels (Fig. 1). In the 2-DE gels, few of the same proteins existed in two or three subcellular fractions, except carbonic anhydrase 2, which indicated that the quality of our subcellular protein extractions was good. The spots identified as the same proteins were primarily located in the vertical or horizontal levels, such as mc7 and mc6, which might be caused by protein degradation, protein modification after translation, or the different structure of proteins. This phenomenon must be further confirmed in future studies.

In this study, most of the altered proteins in microsome and mitochondria mainly participated in disease pathways. Changes in these proteins might affect other metabolic processes and lead to diseases. For example, the catalase activity of lipid peroxidation in microsome and mitochondria was reduced by increased expression and led to prostate disease^33^. In this study, these proteins were changed significantly following BmNPV infection, indicating that these proteins were potentially involved in the response to BmNPV infection. Therefore, subcellular proteomics could not only enhance the resolution of the proteome but also be beneficial for finding proteins relevant to BmNPV resistance.

In the process of BmNPV infection, the virus must penetrate the cytomembrane to enter the intracellular space. Once inside the endosome, nucleocapsids must rely on host cytoskeleton to infect the nucleus and then replicate and assemble within it. In our study, 16 DEPs of interest were identified that were primarily involved in transmembrane transport, viral replication and assembly, energy metabolism, repressing viral infection, and apoptosis.

Endocytosis is an important pathway by which budded viruses (BVs) of baculoviruses enter host cells. This process requires an acidic environment to promote membrane fusion of the BV and endosome to release the nucleocapsid into the cytoplasm^34^,^35^. V-ATPase subunit A is an important transport protein for pH regulation and promotes the infection of baculovirus by acidifying endosomes^36^,^37^,^38^; carbonic anhydrase 2 catalyses the interconversion of carbon dioxide and water to regulate acid balance^39^. These results indicate that resistant silkworm strains may have lower expression levels of V-ATPase subunit A and carbonic anhydrase 2 to repress virus transmembrane transport. In this study, the expression levels of V-ATPase subunit A and carbonic anhydrase 2 in BC9 following BmNPV infection were significantly down-regulated. However, the expression levels of the two proteins did not exhibit differential expression in P50. Once in the cytoplasm, virus require highly conserved host proteins, such as those in the cytoskeleton, to assist transport and reproduction^40^. Tubulin is a major protein constituent of cytoskeletal filaments and is involved in many essential cellular processes, including mitosis, cell motility, and intracellular transport^41^. Fang *et al*. reported that Autographa californica multicapsid nucleopolyhedrovirus (AcMNPV) could be facilitated by interacting with beta-tubulin^42^,^43^. In this study, the expression level of alpha-tubulin was down-regulated more than 6-fold in BC9 following BmNPV infection but was not differentially expressed in P50. Thus, the down-expression of alpha-tubulin indicated its role in nucleocapsids transport in host cells.

After entry into cells, viruses need to rely on the host cell to complete replication. For example, a virus requires the host cell cytoskeleton to assist in transport and assembly, as well as amino acids to synthesize the nucleocapsid^44^,^45^. In this study, several proteins including Tudor-SN, RPS12, CTP1, and PA1 potentially related to viral replication were classified according to relevant references. Tudor-SN is a novel co-activator of E2F-1 that plays essential roles in the G1/S transition during cellular division^46^,^47^. An analysis of intracellular antigens revealed the presence of reovirus in dividing but not quiescent hepatocytes, indicating that cellular division could promote virus replication^48^. RPS12 is a component of the ribosome 40 S subunit that monitors the complementarity of tRNA and mRNA during protein translation. Inoue *et al*. reported that the subunit of CTP1 could ligate to Negri bodies to promote rabies virus transcription and replication^49^,^50^,^51^. The primary function of a proteasome is to degrade unneeded or damaged proteins by proteolysis. Amaya *et al*. reported that this process is used by many viruses to enhance multiplication and sustain persistent infection^52^. According to the PPIs analysis, the CTP1 protein could directly interact with its functional partners Tudor-SN, RPS12, and PA (Fig. 7). Based on their roles in viral replication, the resistant silkworm strain must decrease the expression of these proteins to prevent the replication of a virus, thus explaining the notable down-regulation of the four proteins in BC9 following BmNPV infection without differential expression in P50. Therefore, the decreased expression of these four proteins in the resistant strain highlighted the roles of these proteins in the viral replication process.

Energy metabolism-related proteins were also differentially expressed in BC9 following BmNPV infection. Ndufs8 is a subunit of mitochondrial NADH, which is primarily involved in the binding of two of the six to eight iron-sulfur clusters of a complex. Suszynska-Zajczyk *et al*. reported that Ndufs8 is down-regulated in mice with hyperhomocysteinaemia (HHcy) disease and is involved in energy metabolism^53^. The up-regulation of miR-101 directly repressed herpes simplex virus-1 (HSV-1) replication by negatively regulating ATP5B expression levels^54^. Based on our results, Ndufs8 and ATP5B expression levels in BC9 following BmNPV infection were confirmed to be down-regulated 2.58- and 6.05-fold, respectively, but without notable changes in P50. These two proteins also interacted closely with V-APTase subunit A, CTP1, and alpha-tubulin (Fig. 7). Therefore, we speculated that the host had to decrease energy metabolism to repress BmNPV infection by inhibiting the connection of Ndufs8 and ATP5B with the three proteins mentioned above. PD is a multienzyme complex member that provides the link between glycolysis and the TCA cycle^55^. However, the increased expression level of PD in BC9 following BmNPV infection could not be explained here and requires further study.

Some proteins exhibited up-regulation in BC9 following BmNPV infection, we speculated that these proteins were involved in the resistance to BmNPV infection according to related references. eIF3 plays an important role in promoting mRNA binding to the ribosomal 40 S subunit, which is essential for translation initiation. Gao *et al*. reported that eIF3 could mediate virus resistance in several plant-potyvirus interactions^56^. LDH is an oxidative enzyme that widely exists in cell membranes and cytoplasm and converts lactate into pyruvate during glycolysis. Baba *et al*. reported that LDH expression levels were enhanced following bovine viral diarrhoea virus (BVDV) infection *in vitro*^57^. MSD as the member of aldehyde dehydrogenases plays an important role in repressing white spot syndrome virus replication^58^. We found that these three proteins were not differentially expressed in P50 following BmNPV infection but changed significantly in BC9. Additionally, these proteins could indirectly interact with each other based on the PPI analysis (Fig. 7). Thus, the up-regulation of these proteins in a resistant silkworm strain BC9 indicated that these proteins were involved in response to BmNPV infection.

In addition to the adaptive immune system, apoptosis plays a vital role in resisting virus infection in Lepidopteran insects^10^. In the current study, two proteins related to apoptosis were found in BC9 following BmNPV infection: TPx and PEBP. Powell *et al*. reported that the higher expression of TPx in a scaleless wing mutant of silkworm was responsible for the delayed apoptosis of the scale cells^59^. TPx up-regulation was potentially involved in further repressing viral infection via the apoptosis of infected host cells. PEBP is a novel member of the PEBP family and functions as an anti-apoptotic molecule^60^. In our study, the expression levels of TPx and PEBP in BC9 following BmNPV infection were significantly down-regulated, without notable changes in P50. Therefore, we speculated that the down-regulation of these two proteins could induce enhanced apoptosis to repress the ability of a virus to infect other cells.

In summary, this report is the first to study DEPs in silkworm different resistant strains following BmNPV infection using a comparative subcellular proteomics analysis. Our data provide useful proteomic information of the silkworm midgut response to BmNPV and establish a foundation to clarify the molecular mechanism of silkworm resistance to BmNPV infection.

## Additional Information

**How to cite this article**: Wang, X.-Y. *et al*. Comparative Subcellular Proteomics Analysis of Susceptible and Near-isogenic Resistant *Bombyx mori* (Lepidoptera) Larval Midgut Response to BmNPV infection. *Sci. Rep.* **7**, 45690; doi: 10.1038/srep45690 (2017).

**Publisher's note:** Springer Nature remains neutral with regard to jurisdictional claims in published maps and institutional affiliations.

## Supplementary Material

Supplementary Tables

## Figures and Tables

**Table 1:** The LC_50_ values of different resistant silkworm strains.

Strains	LC_50_ (OB/mL)	95% fiducial limits	
		Lower	Upper
BC9	2.27 × 10^6^	4.58 × 10^5^	1.74 × 10^7^
A35^21^	5.90 × 10^^7^^	2.14 × 10^^7^^	3.22 × 10^^8^^
P50^21^	1.03 × 10^^5^^	3.96 × 10^^4^^	2.24 × 10^^5^^

**Table 2:** Identified proteins from cytosolic fraction that changed significantly in different resistant strains following BmNPV infection.

Spot no.^a^	P50+ vs. P50−b	Ratio^b^	Accession no.^c^	Protein name^c^	Theoretical/Observed P*I*^d^	Theoretical/Observed MW (kDa)^d^	Matched unique peptides^e^	Sequence coverage (%)^e^	Protein score^e^	Molecular/biological function^f^
c1	down	49.13	gi|255652881	Dnaj (Hsp40) homologue 3	5.56/6.47	40/35.25	6	23%	456	Protein folding
c2	down	65.71	gi|512914963	Probable methylmalonate-semialdehyde dehydrogenase [acylating], mitochondrial isoform X1	7.59/7.53	56/57	9	22%	808	Aldehyde dehydrogenase (NAD) activity, fatty-acyl-CoA binding, methylmalonate semialdehyde dehydrogenase (acylating) activity, thymine metabolic process, valine metabolic process
c3	down	149.67	gi|512936895	Acetyl-CoA hydrolase	7.67/7.83	52/48.4	8	16%	523	Hydrolase activity, acetyl-CoA metabolic process
c4	down	6.07	gi|512902782	Uncharacterized protein LOC101738880 isoform X1	5.75/5.72	25/22.73	7	34%	578	
c5	down	5.34	gi|17136564	Alpha-tubulin at 84B [Drosophila melanogaster]	5.00/5.62	51/53.44	8	27%	767	GTPase activity, GTP binding, structural constituent of cytoskeleton, antimicrobial humoral response, mitotic spindle assembly checkpoint
c6	down	2.4	gi|512934077	10 kDa heat shock protein, mitochondrial	6.74/7.38	11/15.22	4	52%	396	ATP binding, protein folding
c7	down	2.86	gi|827563568	Electron transfer flavoprotein subunit alpha, mitochondrial	8.43/7.2	35/29.68	8	36%	827	Electron carrier activity, flavin adenine dinucleotide binding
c8	down	3.72	gi|827558088	3-hydroxyisobutyryl-CoA hydrolase, mitochondrial	8.08/7.43	41/35.39	7	27%	584	Hydrolase activity
c9	down	8.15	gi|512898603	Glyoxylate reductase/hydroxypyruvate reductase-like isoform X1	8.76/7.65	40/36.95	10	33%	849	NAD binding, oxidoreductase activity, acting on the CH-OH group of donors, NAD or NADP as acceptor
c10	down	148.54	gi|112984390	Elongation factor 1-alpha	9.24/6.69	51/49.5	6	18%	390	GTPase activity, GTP binding, translation elongation factor activity
c11	up	3.09	gi|112983556	90-kDa heat shock protein	4.99/5.52	83/86.37	9	16%	713	ATP binding, response to stress, protein folding
c12	up	5.75	gi|512901366	Aldose reductase-like isoform X1	6.09/6.68	36/31.36	8	30%	491	Oxidoreductase activity
c13	up	4.58	gi|827560339	Prolyl endopeptidase	7.90/6.61	90/76.05	4	6%	309	Serine-type endopeptidase activity, serine-type exopeptidase activity
c14	up	3.66	gi|512888904	Cytoplasmic aconitate hydratase-like isoform X1	5.84/6.73	97/94.81	8	13%	55	Metabolic process
c15	up	5.93	gi|512939991	Cystathionine beta-synthase-like	6.02/6.86	54/54.31	8	18%	481	Cystathionine beta-synthase activity, metal ion binding, pyridoxal phosphate binding
c16	up	3.12	gi|357613322	26 S protease regulatory subunit 6 A [Danaus plexippus]	5.11/5.73	48/49.32	7	23%	493	ATP binding, peptidase activity, protein catabolic process
c17	up	3.14	gi|312597598	Inorganic pyrophosphatase	4.96/5.4	32/29.85	9	28%	452	Inorganic diphosphatase activity, magnesium ion binding, phosphate-containing compound metabolic process
c18	up	6.1	gi|512923641	Fatty acid-binding protein-like	5.04/6.16	16/18.89	5	38%	296	Lipid binding, transporter activity
c19	up	4	gi|512923641	Fatty acid-binding protein-like	5.04/6.7	16/16.44	5	33%	200	Lipid binding, transporter activity
c20	up	8.06	gi|512917297	Fatty acid-binding protein 1-like isoform X1	6.59/6.67	15/15.61	7	71%	573	Lipid binding, transporter activity
**Spot no.**^a^	**BC9− vs. P50−**b	**Ratio**^b^	**Accession no.**^c^	**Protein name**^c^	**Theoretical/Observed P*****I***^d^	**Theoretical/Observed MW (kDa)**^d^	**Matched unique peptides**^e^	**Sequence coverage (%)**^e^	**Protein score**^e^	**Molecular/biological function**^f^
c21	down	4.29	gi|512907055	Grpe protein homologue, mitochondrial	6.97/6.2	24/21.21	6	44%	491	Adenyl-nucleotide exchange factor activity, protein folding
c23	down	2.13	gi|114051229	Microtubule-associated protein RP/EB family member 3	5.48/6.27	31/29.98	9	37%	829	
c24	down	2.15	gi|291045214	Isopentenyl-diphosphate delta isomerase	6.37/6.57	30/27.16	8	31%	325	Hydrolase isopentenyl-diphosphate delta-isomerase activity, isoprenoid biosynthetic process
c25	down	2.53	gi|512892238	Carbonic anhydrase 2	5.92/6.41	31/27.45	5	28%	420	Carbonate dehydratase activity, one-carbon metabolic process
c26	down	2.32	gi|160333678	Glutathione S-transferase sigma 2	5.85/6.89	23/24.17	9	53%	864	Transferase activity
c27	down	4.37	gi|112982671	Ribosomal protein S12	5.79/6.21	15/15.97	6	75%	385	Structural constituent of ribosome, translation
c28	up	64.59	gi|49868	Beta-actin (aa 27–375) [Mus musculus]	5.78/6.13	39/32.55	5	19%	389	ATP binding, identical protein binding, kinesin binding, nitric-oxide-synthase binding, RNA polymerase II core promoter proximal region sequence-specific DNA binding
c29	up	72.24	gi|114051866	Isocitrate dehydrogenase	6.24/6.91	47/43.92	7	15%	329	Isocitrate dehydrogenase (NADP+) activity, magnesium ion binding, NAD binding, isocitrate metabolic process, tricarboxylic acid cycle
**c30**	**up**	**2.96**	**gi|153792114**	**Phosphatidylethanolamine-binding protein isoform 2**	**5.96/5.81**	**22/20.57**	**5**	**39%**	**230**	**Defence response to Gram-negative/positive bacteria, regulation of antimicrobial humoral response**
c31	up	9.61	gi|512902782	Uncharacterized protein LOC101738880 isoform X1	5.75/5.55	25/20.54	8	45%	791	
c32	up	4.1	gi|4574740	Tat-binding protein-1 [Drosophila melanogaster]	5.39/5.73	48.4/49.32	3	13%	277	ATPase activity, ATP binding, proteasome-activating ATPase activity, TBP-class protein binding
c33	up	4.88	gi|51555848	Glycerol-3-phosphate dehydrogenase-2	5.62/6.4	39/31.69	10	34%	850	Glycerol-3-phosphate dehydrogenase [NAD+] activity, NAD binding, carbohydrate metabolic process, glycerol-3-phosphate catabolic process
c34	up	2.53	gi|114053311	26 S protease regulatory subunit 6B	5.09/5.61	47/50.98	6	17%	337	ATP binding, peptidase activity, protein catabolic process
c35	up	58.24	gi|347326520	DNA supercoiling factor	4.48/5.53	40/40.96	7	27%	476	Calcium ion binding
**Spot no.^a^**	**BC9+ vs. BC9−b**	**Ratio^b^**	**Accession no.^c^**	**Protein name^c^**	**Theoretical/Observed P*I*^d^**	**Theoretical/Observed MW (kDa)^d^**	**Matched unique peptides^e^**	**Sequence coverage (%)^e^**	**Protein score^e^**	**Molecular/biological function^f^**
c36	down	2.79	gi|512891246	Proteasome subunit alpha type-1 isoform X2	6.01/6.82	31/31.02	8	39%	516	Endopeptidase activity, threonine-type endopeptidase activity
c37	up	136.6	gi|512934137	Selenium-binding protein 1 isoform X2	5.68/6.42	53/52.96	5	15%	298	Selenium binding
c38	up	4.9	gi|512914963	Probable methylmalonate-semialdehyde dehydrogenase [acylating]	7.59/7.36	56/54.89	10	23%	758	Aldehyde dehydrogenase (NAD) activity, fatty-acyl-CoA binding, methylmalonate semialdehyde dehydrogenase (acylating) activity, thymine metabolic process, valine metabolic process
c39	up	10.69	gi|156255210	L-lactate dehydrogenase	6.76/7.72	37/33.93	3	9%	172	L-lactate dehydrogenase activity, carbohydrate metabolic process, carboxylic acid metabolic process

^a^Spot no. corresponding to the numbers in the 2-DE gels of Fig. 2.

^b^The expression intensity ratios of P50 and BC9 following BmNPV infection or strain BC9 to that of P50.

^c^Detailed information on the identified proteins can be viewed via their accession numbers on http://www. ncbi.nlm.nih.gov/.ncbi.nlm.nih.gov/.

^d^Observed molecular weight (MW) and isoelectric point (P*I*) values were obtained from PDQuest analysis. Theoretical MW and P*I* were obtained from a MASCOT analysis.

^e^The three parameters, matched unique peptides, sequence coverage and protein score were generated from a MASCOT analysis.

^f^Molecular/biological functions were annotated using the gene ontology (GO) database (http://www.geneontology.org/).

**Table 3:** Identified proteins from mitochondrial fraction that changed significantly in different resistant strains following BmNPV infection.

Spot no.^a^	P50+ vs. P50−^b^	Ratio^b^	Accession no^c^	Protein name^c^	Theoretical/Observed P*I*^d^	Theoretical/Observed MW (kDa)^d^	Pep. Count^e^	Sequence coverage (%)^e^	Protein Score^e^	Molecular/biological function^f^
mc1	down	4.23	gi|62241292	Protein disulfide-isomerase	5.3/5.75	55.5/52.39	17		99.97	Protein disulfide isomerase activity, cell redox homeostasis
mc2	down	11.58	gi|336454478	Heat shock protein 70–3	5.12/6.9	72.8/30.53	17		100	ATP binding
mc3	down	6.31	gi|827547570	Dihydrolipoyllysine-residue succinyltransferase component of 2-oxoglutarate dehydrogenase complex	9.28/6.93	50.4/53.97	7	18%	361/58	Dihydrolipoyllysine-residue succinyltransferase activity, tricarboxylic acid cycle
mc4	up	12.41	gi|114052454	NADH dehydrogenase [ubiquinone] 1 beta subcomplex subunit 10	5.93/6.62	19.3/17.96	8	60%	521/58	Oxidation-reduction process
mc5	up	17.81	gi|512890394	Golgin subfamily A member 4	5.05/6.78	30.8/17.3	43		95.118	Protein targeting to Golgi
mc6	up	11.56	gi|512928976	Voltage-dependent anion-selective channel isoform X2	6.96/6.79	30.1/27.03	18		100	Voltage-gated anion channel activity
mc7	up	3.65	gi|512928976	Voltage-dependent anion-selective channel isoform X2	6.96/7.42	30.1/27.03	17		100	Voltage-gated anion channel activity
mc8	up	5.02	gi|98990259	Cytochrome b-c1 complex subunit Rieske	8.59/7.25	29.4/21.47	11		99.982	2 iron, 2 sulfur cluster binding, metal ion binding, ubiquinol-cytochrome-c reductase activity
**Spot no.**^**a**^	**BC9− vs. P50−**^**b**^	**Ratio**^**b**^	**Accession no.**^**c**^	**Protein name**^**c**^	**Theoretical/Observed P*****I***^**d**^	**Theoretical/Observed MW (kDa)**^**d**^	**Matched unique peptides**^**e**^	**Pep. Count**^**e**^	**PROTEIN score(%)**^**e**^	**Molecular/biological function**^**f**^
**mc9**	**up**	**2.58**	**gi|87248369**	**NADH dehydrogenase (ubiquinone) Fe-S protein 8**	**6.15/5.79**	**25.5/21.83**	**13**		**100**	**4 iron, 4 sulfur cluster binding, oxidoreductase activity, acting on NAD(P)H**
mc10	up	3.86	gi|38260562	Thiol peroxiredoxin	6.09/7	22.07/19.63	10		100	Peroxiredoxin activity
mc11	up	3.62	gi|98990259	Cytochrome b-c1 complex subunit Rieske	8.59/7.25	29.4/21.47	11		99.982	2 iron, 2 sulfur cluster binding, metal ion binding, ubiquinol cytochrome c reductase activity
**Spot no.**^**a**^	**BC9+ vs. BC9−**^**b**^	**Ratio**^**b**^	**Accession no.**^**c**^	**Protein name**^**c**^	**Theoretical/Observed P*****I***^**d**^	**Theoretical/Observed MW (kDa)**^**d**^	**Matched unique peptides**^**e**^	**Pep. Count**^**e**^	**Protein score(%)**^**e**^	**Molecular/biological function**^**f**^
mc12	down	6.05	gi|95102940	H^+^-transporting ATP synthase beta subunit isoform 2	5.32/5.92	54.9/47	15		99.488	ATP binding, proton-transporting ATP synthase activity
mc13	down	4.44	gi|87248085	Chaperonin-containing t-complex polypeptide 1 beta	6.32/6.96	58/55.19	18		100	ATP binding, protein folding
mc14	up	2.1	gi|512892238	Carbonic anhydrase 2	5.92/6.31	30.04/26.15	9		100	Carbonate dehydratase activity, one-carbon metabolic process

a, Spot no. corresponds to the numbers in the 2-DE gels of Fig. 3.b,c,d,e,f are the same as in Table 2.

**Table 4:** Identified proteins from microsomal fraction that changed significantly in different resistant strains following BmNPV infection.

Spot no.^a^	P50+ vs. P50−^b^	Ratio^b^	Accession no.^c^	Protein name^c^	Theoretical/Observed P*I*^d^	Theoretical/Observed MW (kDa)^d^	Matched unique peptides^e^	Sequence coverage (%)^e^	Protein score^e^	Molecular/biological function^f^
ms1	down	33.35	gi|148298800	Enolase	5.62/6.1	47/47.82	9	30%	795	Magnesium ion binding, phosphopyruvate hydratase complex, glycolytic process
ms2	down	27.28	gi|512913423	Uncharacterized protein LOC101745964	6.04/6.02	51/53	4	11%	193	
ms3	down	78.57	gi|112983322	Transitional endoplasmic reticulum ATPase TER94	5.3/5.94	90/101.55	8	15%	589	ATPase activity, hydrolase activity
ms4	down	48.18	gi|114051800	Eukaryotic translation initiation factor 3 subunit I	5.71/6.39	37/36.77	10	42%	707	Translation initiation factor activity, formation of translation preinitiation complex, regulation of translational initiation
ms5	down	13.14	gi|512892238	Carbonic anhydrase 2	5.92/6.39	31/28.44	4	22%	265	Carbonate dehydratase activity, one-carbon metabolic process
ms6	down	65.11	gi|512912927	Sorting nexin lst-4	5.61/6.4	64/71.17	9	17%	415	Phosphatidylinositol-3,4,5-trisphosphate binding, intracellular protein transport, phagosome-lysosome fusion involved in apoptotic cell clearance
ms7	down	15.99	gi|114053117	Eukaryotic translation initiation factor 3 subunit K	5.38/6	25/24.27	3	12%	170	Ribosome binding, translation initiation factor activity, formation of translation preinitiation complex, regulation of translational initiation
ms8	down	2.83	gi|112983906	Eukaryotic translation initiation factor 3 subunit H	5.68/5.92	39/54.61	5	16%	343	Translation initiation factor activity, formation of translation preinitiation complex
ms9	down	9.05	gi|112983898	Elongation factor 1 gamma	5.83/6.47	49/46.85	8	21%	587	Translation elongation factor activity
ms10	down	2.68	gi|112983010	Translation elongation factor 2 isoform 1	6.23/7.16	98/113.15	4	9%	223	GTPase activity, GTP binding, translation elongation factor activity
ms11	up	2.5	gi|827548126	Pyruvate dehydrogenase E1 component beta subunit isoform X1	6.03/6.04	40/32.43	10	35%	594	Pyruvate dehydrogenase (acetyl-transferring) activity, acetyl-CoA biosynthetic process from pyruvate
ms12	up	15.59	gi|87248109	Enoyl-CoA hydratase precursor 1	8.44/6.72	32/26.8	7	31%	452	Catalytic activity
ms13	up	9.14	gi|827537214	Probable enoyl-CoA hydratase, mitochondrial	9.28/7.14	32/26.74	10	42%	874	Catalytic activity
ms14	up	3.08	gi|827537214	Probable enoyl-CoA hydratase, mitochondrial	9.28/7.69	32/26.84	10	39%	896	Catalytic activity
ms15	up	2.09	gi|114052278	ATP synthase	9.21/6.91	60/51.66	4	5%	128	ATP binding, proton-transporting ATPase activity, proton-transporting ATP synthase activity
ms16	up	3.87	gi|153792309	Pyruvate dehydrogenase	8.07/7.43	44/34.96	8	27%	410	Pyruvate dehydrogenase (acetyl-transferring) activity, glycolytic process
**Spot no.**^**a**^	**BC9− vs. P50−**^**b**^	**Ratio**^**b**^	**Accession no.**^**c**^	**Protein name**^**c**^	**Theoretical/Observed P*****I***^**d**^	**Theoretical/Observed MW (kDa)**^**d**^	**Matched unique peptides**^**e**^	**Sequence coverage (%)**^**e**^	**Protein score**^**e**^	**Molecular/biological function**^**f**^
ms17	down	99.96	gi|512903088	Mitochondrial import receptor subunit Tom70	5.55/6.31	62/62.95	8	16%	391	Receptor
ms18	down	5.77	gi|512899307	Esterase FE4-like	5.27/6.12	68/66.96	5	5%	206	Hydrolase activity
ms19	down	64.93	gi|112983574	Carboxylic ester hydrolase	7.09/6.85	55/71.74	6	16%	217	Hydrolase activity
ms20	up	166.38	gi|336454478	Heat shock protein 70–3	5.12/5.59	73/71.7	10	21%	647	ATP binding
**ms21**	**up**	**65.6**	**gi|304307739**	**Tudor staphylococcus/micrococcal nuclease**	**8.56/6.12**	**99/26.35**	**9**	**13%**	**664**	**Transcription cofactor activity, posttranscriptional gene silencing by RNA**
ms22	up	38.05	gi|112983926	Arginine kinase	5.87/6.75	40/42.06	9	32%	561	ATP binding, kinase activity,
ms23	up	33.53	gi|124245114	Glucose-regulated protein 78 [Fenneropenaeus chinensis]	5.00/6.74	72.8/48.85	5	11%	491	ATP binding, Nucleotide-binding
ms24	up	34.91	gi|112982960	Ferritin precursor	6.75/7.56	26/26.95	4	31%	441	Ferric iron binding, ferroxidase activity, cellular iron ion homeostasis, iron ion transport
ms25	up	8	gi|153792257	Trypsin-like protease	5.62/6.5	28/25.84	3	17%	184	Serine-type endopeptidase activity
ms26	up	10.16	gi|5751	Actin a3	5.47/6.05	42/68.45	4	12%	238	ATP binding
ms27	up	2.5	gi|827537214	Probable enoyl-CoA hydratase, mitochondrial	9.28/7.69	32/26.84	10	39%	893	Catalytic activity
**Spot no.^a^**	**BC9+ vs. BC9−^b^**	**Ratio^b^**	**Accession no.^c^**	**Protein name^c^**	**Theoretical/Observed P*I*^d^**	**Theoretical/Observed MW (kDa)^d^**	**Matched unique peptides^e^**	**Sequence coverage (%)^e^**	**Protein score^e^**	**Molecular/biological function^f^**
ms28	down	2.06	gi|827548126	Pyruvate dehydrogenase e1 component beta subunit isoform x1	6.03/6.04	40/32.43	10	35%	625	Pyruvate dehydrogenase (acetyl-transferring) activity, acetyl-CoA biosynthetic process from pyruvate
ms29	down	6.04	gi|32400724	Alpha-tubulin [oikopleura dioica]	4.94/5.64	51/55.62	9	31%	805	GTPase activity, GTP binding, structural constituent of cytoskeleton
ms30	down	2.76	gi|148298878	Vacuolar ATP synthase catalytic subunit a	5.27/6.18	69/58.88	9	16%	622	ATP binding, proton-transporting ATPase activity, ATP hydrolysis-coupled proton transport, ATP metabolic process
ms31	down	2.3	gi|15213812	Ribosomal protein s12 [spodoptera frugiperda]	5.79/6.17	15/15.67	6	62%	542	Structural constituent of ribosome, translation
ms32	up	198.68	gi|153092309	Pyruvate dehydrogenase	8.07/7.43	44/34.96	9	28%	511	Pyruvate dehydrogenase (acetyl-transferring) activity, glycolytic process
ms33	up	2.47	gi|112982996	Thiol peroxiredoxin	6.09/7.13	22/20.17	6	37%	456	Peroxiredoxin activity
ms34	up	5.79	gi|112983898	Elongation factor 1 gamma	5.83/6.48	49/46.85	10	26%	704	Translation elongation factor activity
ms35	up	3.4	gi|114051800	Eukaryotic translation initiation factor 3 subunit I	5.71/6.52	37/36.46	10	32%	672	Translation initiation factor activity, formation of translation preinitiation complex, regulation of translational initiation

a, Spot no. corresponds to the numbers in the 2-DE gels of Fig. 4.b,c,d,e,f are the same as in Table 2.

## References

[b1] Goldsmith M. R., Shimada T. & Abe H. The genetics and genomics of the silkworm, Bombyx mori. Annu Rev Entomol. 50, 71–100 (2005). 15355234 10.1146/annurev.ento.50.071803.130456

[b2] Shao Q. M. . Hindgut Innate Immunity and Regulation of Fecal Microbiota through Melanization in Insects. J Biol Chem. 287, 14270–14279 (2012). 22375003 10.1074/jbc.M112.354548 PMC3340165

[b3] Bao Y. Y. . Gene expression profiling of resistant and susceptible *Bombyx mori* strains reveals nucleopolyhedrovirus-associated variations in host gene transcript levels. Genomics. 94, 138–145 (2009). 19389468 10.1016/j.ygeno.2009.04.003

[b4] Nakazawa H. . Antiviral activity of a serine protease from the digestive juice of *Bombyx mori* larvae against nucleopolyhedrovirus. Virology. 321, 154–162 (2004). 15033574 10.1016/j.virol.2003.12.011

[b5] Ponnuvel K. M. . A lipase isolated from the silkworm *Bombyx mori* shows antiviral activity against nucleopolyhedrovirus. J Virol. 77, 10725–10729 (2003). 12970462 10.1128/JVI.77.19.10725-10729.2003 PMC228431

[b6] Ponnuvel K. M., Nithya K., Sirigineedi S., Awasthi A. K. & Yamakawa M. *In Vitro* Antiviral Activity of an Alkaline Trypsin from the Digestive Juice of *Bombyx Mori* Larvae against Nucleopolyhedrovirus. Arch Insect Biochem Physiol. 81, 90–104 (2012). 22898997 10.1002/arch.21046

[b7] Selot R. . Identification of a soluble NADPH oxidoreductase (BmNOX) with antiviral activities in the gut juice of *Bombyx mori*. Biosci Biotechnol Biochem. 71, 200–5 (2007). 17213661 10.1271/bbb.60450

[b8] Kang L. Q. . Arginine kinase is highly expressed in a resistant strain of silkworm (*Bombyx mori*, Lepidoptera): Implication of its role in resistance to *Bombyx mori* nucleopolyhedrovirus. Comp Biochem Physiol Biochem Mol Biol. 158, 230–234 (2011). 10.1016/j.cbpb.2010.12.001 21146627

[b9] Liu X., Yao Q., Wang Y. & Chen K. Proteomic analysis of nucleopolyhedrovirus infection resistance in the silkworm, *Bombyx mori* (Lepidoptera: Bombycidae). J Invertebr Pathol. 105, 84–90 (2010). 20471391 10.1016/j.jip.2010.05.007

[b10] Cheng Y., Wang X. Y., Hu H., Killiny N. & Xu J. P. A Hypothetical Model of Crossing *Bombyx mori* Nucleopolyhedrovirus through Its Host Midgut Physical Barrier. PLoS One (2014). 10.1371/journal.pone.0115032 PMC4264868 25502928

[b11] Stekhoven D. J., Omasits U., Quebatte M., Dehio C. & Ahrens C. H. Proteome-wide identification of predominant subcellular protein localizations in a bacterial model organism. J Proteomics. 99, 123–137 (2014). 24486812 10.1016/j.jprot.2014.01.015

[b12] Khan R. . Protein expression profiling of nuclear membrane protein reveals potential biomarker of human hepatocellular carcinoma. Clin Proteomics. 10, 6 (2013). 23724895 10.1186/1559-0275-10-6 PMC3691657

[b13] Schumacker P. T. . Mitochondria in lung biology and pathology: more than just a powerhouse. Am J Physiol Lung Cell Mol Physiol. 306, L962–L974 (2014). 24748601 10.1152/ajplung.00073.2014 PMC4042189

[b14] Wu X. . Mitochondrial proteomic analysis of human host cells infected with H3N2 swine influenza virus. J Proteomics. 91, 136–50 (2013). 23856606 10.1016/j.jprot.2013.06.037

[b15] Sofra V. . Antigen-loaded ER microsomes from APC induce potent immune responses against viral infection. Eur J Immunol. 39, 85–95 (2009). 19089809 10.1002/eji.200838443

[b16] Wang S. Q. . Imaging microdomain Ca^2+^ in muscle cells. Circ Res. 94, 1011–22 (2004). 15117829 10.1161/01.RES.0000125883.68447.A1

[b17] Perham R. N. Swinging arms and swinging domains in multifunctional enzymes: Catalytic machines for multistep reactions. Annu Rev Biochem. 69, 961–1004 (2000). 10966480 10.1146/annurev.biochem.69.1.961

[b18] Nandi D., Tahiliani P., Kumar A. & Chandu D. The ubiquitin-proteasome system. J Biosci. 31, 137–55 (2006). 16595883 10.1007/BF02705243

[b19] Yao Q. . Screening of molecular markers for NPV resistance in *Bombyx mori* L. (Lep., Bombycidae). J. Appl. Entomol. 127, 134–136 (2003).

[b20] Rahman M. M. & Gopinathan K. P. Systemic and *in vitro* infection process of *Bombyx mori* nucleopolyhedrovirus. Virus Res. 101, 109–18 (2004). 15041178 10.1016/j.virusres.2003.12.027

[b21] Cheng Y., Wang X. Y., Du C., Gao J. & Xu J. P. Expression analysis of several antiviral related genes to BmNPV in different resistant strains of silkworm, *Bombyx mori*. J Insect Sci. 14 (2014). 10.1093/jis/14.1.76 PMC4212868 25373223

[b22] Qin Y., Lu G., Keping C. & Zhigang H. Detection of proliferation of *Bombyx mori* nucleopolyhedrovirus in its host by fluorescence quantitative PCR. Acta Entomologica Sinica. 48, 871–875 (2005).

[b23] Lu Z. J., Hu H. & Killiny N. Proteomic maps of subcellular protein fractions of the Asian citrus psyllid *Diaphorina citri*, the vector of citrus huanglongbing. *Physiol Entomol*. (2016).24. Ivashov, V.A. *et al*. Lipidome and proteome of lipid droplets from the methylotrophic yeast *Pichia pastoris*. Biochim Biophys Acta. 1831, 282–290 (2013). 23041514 10.1016/j.bbalip.2012.09.017 PMC3787741

[b24] Ivashov V. A. . Lipidome and proteome of lipid droplets from the methylotrophic yeast *Pichia pastoris*. Biochim Biophys Acta. 1831, 282–290 (2013). 23041514 10.1016/j.bbalip.2012.09.017 PMC3787741

[b25] Bradford M. M. A rapid and sensitive method for the quantitation of microgram quantities of protein utilizing the principle of protein-dye binding. Anal Biochem. 72, 248–54 (1976). 942051 10.1016/0003-2697(76)90527-3

[b26] Lu Z. J. . Proteomic analysis of the host response in the bursa of Fabricius of chickens infected with Marek’s disease virus. Virus Res. 153, 250–257 (2010). 20723570 10.1016/j.virusres.2010.08.010

[b27] Steiner S. . Proteomics to display lovastatin-induced protein and pathway regulation in rat liver. Electrophoresis. 21, 2129–37 (2000). 10892724 10.1002/1522-2683(20000601)21:11<2129::AID-ELPS2129>3.0.CO;2-V

[b28] Barabasi A. L., Gulbahce N. & Loscalzo J. Network medicine: a network-based approach to human disease. Nat Rev Genet. 12, 56–68 (2011). 21164525 10.1038/nrg2918 PMC3140052

[b29] Jensen L. J. . STRING 8--a global view on proteins and their functional interactions in 630 organisms. Nucleic Acids Res. 37, D412–6 (2009). 18940858 10.1093/nar/gkn760 PMC2686466

[b30] Zhou Z. H. . Comparative Proteomic Analysis between the Domesticated Silkworm (*Bombyx mori*) Reared on Fresh Mulberry Leaves and on Artificial Diet. J Proteome Res. 7, 5103–5111 (2008). 18998723 10.1021/pr800383r

[b31] Huber L. A., Pfaller K. & Vietor I. Organelle proteomics: implications for subcellular fractionation in proteomics. Circ Res. 92, 962–8 (2003). 12750306 10.1161/01.RES.0000071748.48338.25

[b32] Pradet-Balade B., Boulme F., Beug H., Mullner E. W. & Garcia-Sanz J. A. Translation control: bridging the gap between genomics and proteomics? Trends Biochem Sci. 26, 225–229 (2001). 11295554 10.1016/s0968-0004(00)01776-x

[b33] Kvitsinadze N. . Peroxidation processes in mitochondria and microsome of human prostate tissues at different pathology. Eur Urol. 8, 616–616 (2009).

[b34] Long G., Pan X. Y., Kormelink R. & Vlak J. M. Functional entry of baculovirus into insect and mammalian cells is dependent on clathrin-mediated endocytosis. J Virol. 80, 8830–8833 (2006). 16912330 10.1128/JVI.00880-06 PMC1563848

[b35] Kingsley D. H., Behbahani A., Rashtian A., Blissard G. W. & Zimmerberg J. A discrete stage of baculovirus GP64-mediated membrane fusion. Mol Biol Cell. 10, 4191–200 (1999). 10588652 10.1091/mbc.10.12.4191 PMC25752

[b36] Hinton A., Bond S. & Forgac M. V-ATPase functions in normal and disease processes. Pflugers Arch. 457, 589–98 (2009). 18026982 10.1007/s00424-007-0382-4

[b37] Beyenbach K. W. & Wieczorek H. The V-type H+ ATPase: molecular structure and function, physiological roles and regulation. J Exp Biol. 209, 577–89 (2006). 16449553 10.1242/jeb.02014

[b38] Forgac M. Vacuolar ATPases: rotary proton pumps in physiology and pathophysiology. Nat Rev Mol Cell Biol. 8, 917–29 (2007). 17912264 10.1038/nrm2272

[b39] Lin T. Y. . Carbonic anhydrase 2-like a and 15a are involved in acid-base regulation and Na+ uptake in zebrafish H+-ATPase-rich cells. Am J Physiol Cell Physiol. 294, C1250–60 (2008). 18322140 10.1152/ajpcell.00021.2008

[b40] Volkman L. E. Baculovirus infectivity and the actin cytoskeleton. Curr Drug Targets. 8, 1075–83 (2007). 17979667 10.2174/138945007782151379

[b41] Gunning P. W., Ghoshdastider U., Whitaker S., Popp D. & Robinson R. C. The evolution of compositionally and functionally distinct actin filaments. J Cell Sci. 128, 2009–19 (2015). 25788699 10.1242/jcs.165563

[b42] Fang M. G., Nie Y. C. & Theilmann D. A. AcMNPV EXON0 (AC141) which is required for the efficient egress of budded virus nucleocapsids interacts with beta-tubulin. Virology. 385, 496–504 (2009). 19155039 10.1016/j.virol.2008.12.023

[b43] Jolly C., Mitar I. & Sattentau Q. J. Requirement for an intact T-cell actin and tubulin cytoskeleton for efficient assembly and spread of human immunodeficiency virus type 1. J Virol. 81, 5547–5560 (2007). 17360745 10.1128/JVI.01469-06 PMC1900271

[b44] Radtke K., Dohner K. & Sodeik B. Viral interactions with the cytoskeleton: a hitchhiker’s guide to the cell. Cell Microbiol. 8, 387–400 (2006). 16469052 10.1111/j.1462-5822.2005.00679.x

[b45] Guo Z. J. . Characterization of aggregate/aggresome structures formed by polyhedrin of *Bombyx mori* nucleopolyhedrovirus. Sci Rep. 5, 14601 (2015). 26440217 10.1038/srep14601 PMC4594129

[b46] Fashe T., Saarikettu J., Isomaki P., Yang J. & Silvennoinen O. Expression analysis of Tudor-SN protein in mouse tissues. Tissue Cell. 45, 21–31 (2013). 23068188 10.1016/j.tice.2012.09.001

[b47] Su C. . Tudor Staphylococcal Nuclease (Tudor-SN), a Novel Regulator Facilitating G(1)/S Phase Transition, Acting as a Co-activator of E2F-1 in Cell Cycle Regulation. J Biol Chem. 290, 7208–7220 (2015). 25627688 10.1074/jbc.M114.625046 PMC4358140

[b48] Taterka J., Sutcliffe M. & Rubin D. H. Selective reovirus infection of murine hepatocarcinoma cells during cell division. A model of viral liver infection. J Clin Invest. 94, 353–60 (1994). 8040276 10.1172/JCI117329 PMC296316

[b49] Inoue Y. . Chaperonin TRiC/CCT participates in replication of hepatitis C virus genome via interaction with the viral NS5B protein. Virology. 410, 38–47 (2011). 21093005 10.1016/j.virol.2010.10.026

[b50] Zhang J. Y. . Cellular Chaperonin CCT gamma Contributes to Rabies Virus Replication during Infection. J Virol. 87, 7608–7621 (2013). 23637400 10.1128/JVI.03186-12 PMC3700271

[b51] Zhang J. Y. . The chaperonin CCT alpha is required for efficient transcription and replication of rabies virus. Microbiol Immunol. 58, 590–599 (2014). 25082455 10.1111/1348-0421.12186

[b52] Amaya M. . The Ubiquitin Proteasome System Plays a Role in Venezuelan Equine Encephalitis Virus Infection. PLoS One. 10, (2015). 10.1371/journal.pone.0124792 PMC4415917 25927990

[b53] Suszynska-Zajczyk J., Sikora M. & Jakubowski H. Paraoxonase 1 deficiency and hyperhomocysteinemia alter the expression of mouse kidney proteins involved in renal disease. Mol Genet Metab. 113, 200–206 (2014). 25069821 10.1016/j.ymgme.2014.07.011

[b54] Zheng S. Q., Li Y. X., Zhang Y., Li X. & Tang H. MiR-101 regulates HSV-1 replication by targeting ATP5B. Antiviral Res. 89, 219–226 (2011). 21291913 10.1016/j.antiviral.2011.01.008

[b55] Bhandary S. & Aguan K. Pyruvate dehydrogenase complex deficiency and its relationship with epilepsy frequency–An overview. Epilepsy Res. 116, 40–52 (2015). 26354166 10.1016/j.eplepsyres.2015.07.002

[b56] Gao L.. NIa-Pro of *Papaya ringspot virus* interacts with *Carica papaya* eukaryotic translation initiation factor 3 subunit G (CpeIF3G). Virus Genes. 50, 97–103 (2015). 25416301 10.1007/s11262-014-1145-x

[b57] Baba C., Yanagida K., Kanzaki T. & Baba M. Colorimetric lactate dehydrogenase (LDH) assay for evaluation of antiviral activity against bovine viral diarrhoea virus (BVDV) *in vitro*. Antivir Chem Chemother. 16, 33–9 (2005). 15739620 10.1177/095632020501600104

[b58] Lin Y. R. . The Role of Aldehyde Dehydrogenase and Hsp70 in Suppression of White Spot Syndrome Virus Replication at High Temperature. J Virol. 85, 3517–3525 (2011). 21228234 10.1128/JVI.01973-10 PMC3067880

[b59] Shi X. F. . Proteomic analysis of the phenotype of the scaleless wings mutant in the silkworm, *Bombyx mori*. J Proteomics. 78, 15–25 (2013). 23174119 10.1016/j.jprot.2012.11.003

[b60] Li H. . Phosphatidylethanolamine-binding protein 4 is associated with breast cancer metastasis through Src-mediated Akt tyrosine phosphorylation. Oncogene. 33, 4589–4598 (2014). 24276246 10.1038/onc.2013.408

