# Supplementary material for: Comparative Subcellular Proteomics Analysis of Susceptible and Near-isogenic Resistant Bombyx mori (Lepidoptera) Larval Midgut Response to BmNPV infection
Source: Sci Rep. 2017 Mar 31;7:45690. doi: 10.1038/srep45690 (PMC5374506; doi:10.1038/srep45690)

Comparative Subcellular Proteomics Analysis of Susceptible and Near-isogenic Resistant *Bombyx mori* (Lepidoptera) Larval Midgut Response to BmNPV infection

Xue-yang Wang[1], Hai-zhong Yu[1], Jia-ping Xu[1]*, Shang-zhi Zhang[1], Dong Yu[1], Ming-hui Liu[2], Lin-ling Wang[3]

Table S1. Primers used in RT-qPCR for validation of selected proteins.

Gene name	Forward primers (5'-3')	Reverse primers (5'-3')
Tudor staphylococcus/micrococcal nuclease (Tudor-SN)	TCCAAGGAAGGGAGCCATCTG	GGACCTGCCTATTGTCTGTTATTT
Alpha-tubulin	CAACTACCAGCCACCAACCG	ACACCGACCTCTTCGTAATCCTT
Vacuolar ATP synthase catalytic subunit A (V-ATPase subunit A)	AGTTCAAAATGGCGAGCAAAG	CGACGGGTCCAGATACGG
Ribosomal protein S12 (RpS12)	TTGAGCGGAGGTGCTATGGAT	CACAAGTGGGATTTGATGTTCGT
Pyruvate dehydrogenase (PD)	CTGTATTCAGGACAAGAAGCCGT	CCCCAGCACACTAACACCCAT
Thiol peroxiredoxin (TPx)	CCCTTCCGAGGACTCTTCATCA	GTCGGGCTTGATGGTCTTGG
Eukaryotic translation initiation factor 3 subunit I (eIF3)	AGGACTGGTAAAAAAATCCACTCTG	GGTTGTCACTTCCATAGCATCTTG
NADH dehydrogenase (ubiquinone) Fe-S protein 8 (Ndufs8)	TGGTGTGATAGTGAAACGGGC	TTGATTGTTGCTGGTTCCTTGA
H+ transporting ATP synthase beta subunit isoform 2 (ATP5B)	GAAACCCTCGGTCGCATCAT	TCCGCCAAACAACCCAATCT
Chaperonin containing t-complex polypeptide 1 beta (CTP1)	ACATTCTGAAGGCAGCACCC	TCGGCAAGCAAGTTTTAGGC
Carbonic anhydrase 2	ACAAGGTCTGGAGGGGATTCA	GGTGTAGGGCGGTGTCGTC
Phosphatidylethanolamine binding protein isoform 2 (PEBP)	TGAAATACCCAAGCGGAGTCG	GGTTCTTTACGGGACGGCG
Proteasome subunit alpha type-1 isoform X2 (PA1)	AGACTTGTCCATCAGCCAACTACTT	GAGATAACTCTTCCTCGTCGCAT
Selenium-binding protein 1 isoform X2 (SB1)	GCTTGTATGTCTCGTCGTCGC	ATGCCTCCATTCACAGTATCCAC
Methylmalonate-semialdehyde dehydrogenase (MSD)	CGCCGAAATAACTCTCCCCA	TCTTGCGGTTCCTTTGGTCAT
L-lactate dehydrogenase (LDH)	CATCCGCCAGCCTCTAACC	ACTCCGCTCAACGCACCA
BmGAPDH	CCGCGTCCCTGTTGCTAAT	CTGCCTCCTTGACCTTTTGC

Table S2. The expression patterns of DEGs in different pathways following BmNPV infection

			Microsome	Mitochondria	Cytosol	P50+ vs. P50-		BC9- vs. P50-		BC9+ vs. BC9-	
						up	down	up	down	up	down
	Glycolysis/Gluconeogenesis	ko00010	5	0	2	2	1	0	0	2	2
	Peroxisome	ko04146	5	0	1	3	0	3	0	0	0
	Citrate cycle (TCA cycle)	ko00020	4	1	2	3	1	1	0	0	2
Enegy metabolism	Oxidative phosphorylation	ko00190	4	4	1	4	0	3	1	1	0
	Pyruvate metabolism	ko00620	4	0	4	2	2	0	0	2	2
	Carbon metabolism	ko01200	4	1	5	3	4	1	0	1	2
	Propanoate metabolism	ko00640	0	0	4	0	2	0	0	2	0
Total number						17	8	8	1	6	8
	Protein processing in endoplasmic reti	ko04141	3	4	2	1	5	2	0	1	0
	Biosynthesis of amino acids	ko01230	1	0	3	2	1	1	0	0	0
Protein metabolism	Valine, leucine and isoleucine degrada	ko00280	0	0	4	0	2	0	0	2	0
	beta-Alanine metabolism	ko00410	0	0	4	0	2	0	0	2	0
	Proteasome	ko03050	0	0	4	1	0	2	0	0	1
Total number						4	10	5	0	5	1
	Glucagon signaling pathway	ko04922	4	0	1	2	0	0	0	1	2
Signaling pathway	Collecting duct acid secretion	ko04966	3	0	0	0	0	1	1	1	0
	PPAR signaling pathway	ko03320	0	0	3	3	0	0	0	0	0
Total number						5	0	1	1	2	2
	Renal cell carcinoma	ko04066	5	0	0	2	1	0	0	0	2
	Legionellosis	ko05134	5	1	1	0	4	2	0	1	0
	Vibrio cholerae infection	ko05110	4	0	0	0	0	3	1	1	0
	Central carbon metabolism in cancer	ko05230	4	0	0	2	0	0	0	0	2
	Epithelial cell signaling in Helicobact	ko05120	3	0	0	0	0	0	1	1	0
	Measles	ko05162	3	1	0	0	2	2	0	0	0
Human disease	Influenza A	ko05164	3	1	1	0	1	4	0	0	0
	Epstein-Barr virus infection	ko05169	3	1	3	1	1	5	0	0	0
	Rheumatoid arthritis	ko05323	3	0	0	1	1	0	0	1	0
	Alzheimer's disease	ko05010	1	4	0	3	0	2	0	0	0

	Prion diseases	ko05012	1	7	0	5	1	2	0	0	0
	Huntington's disease	ko05016	1	6	0	5	0	2	0	0	0
	Non-alcoholic fatty liver disease (NAI	ko04932	0	4	0	2	0	2	0	0	0
Total number						21	11	24	2	4	4
Transport	Phagosome	ko04145	5	0	2	0	1	3	0	1	2
	Endocytosis	ko04144	3	3	0	0	2	2	0	0	2
	Synaptic vesicle cycle	ko04721	3	0	0	0	0	1	1	1	2
	RNA transport	ko03013	3	0	1	0	3	0	0	1	0
Total number						0	6	6	1	3	6
Digestion	Primary bile acid biosynthesis	ko00120	5	0	0	0	3	2	0	0	0
Total number						0	3	2	0	0	0

Figure S1. The full-length gel of Fig. 1.

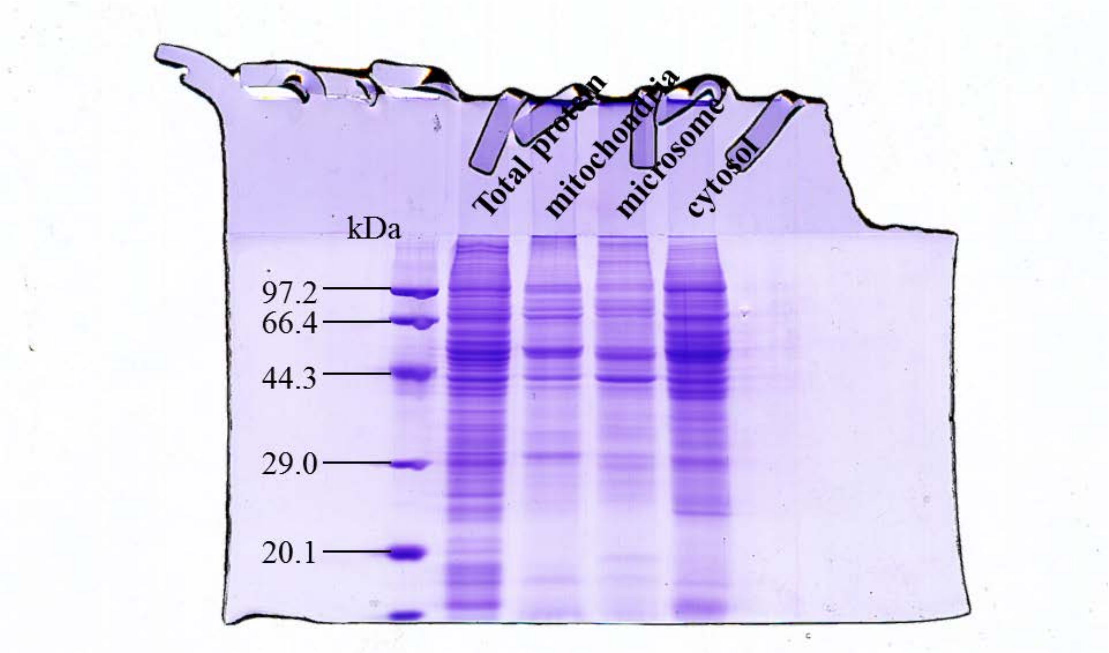

Figure S1. The full-length gels of cytosol in P50 and BC9 following BmNPV infection.

P50-:

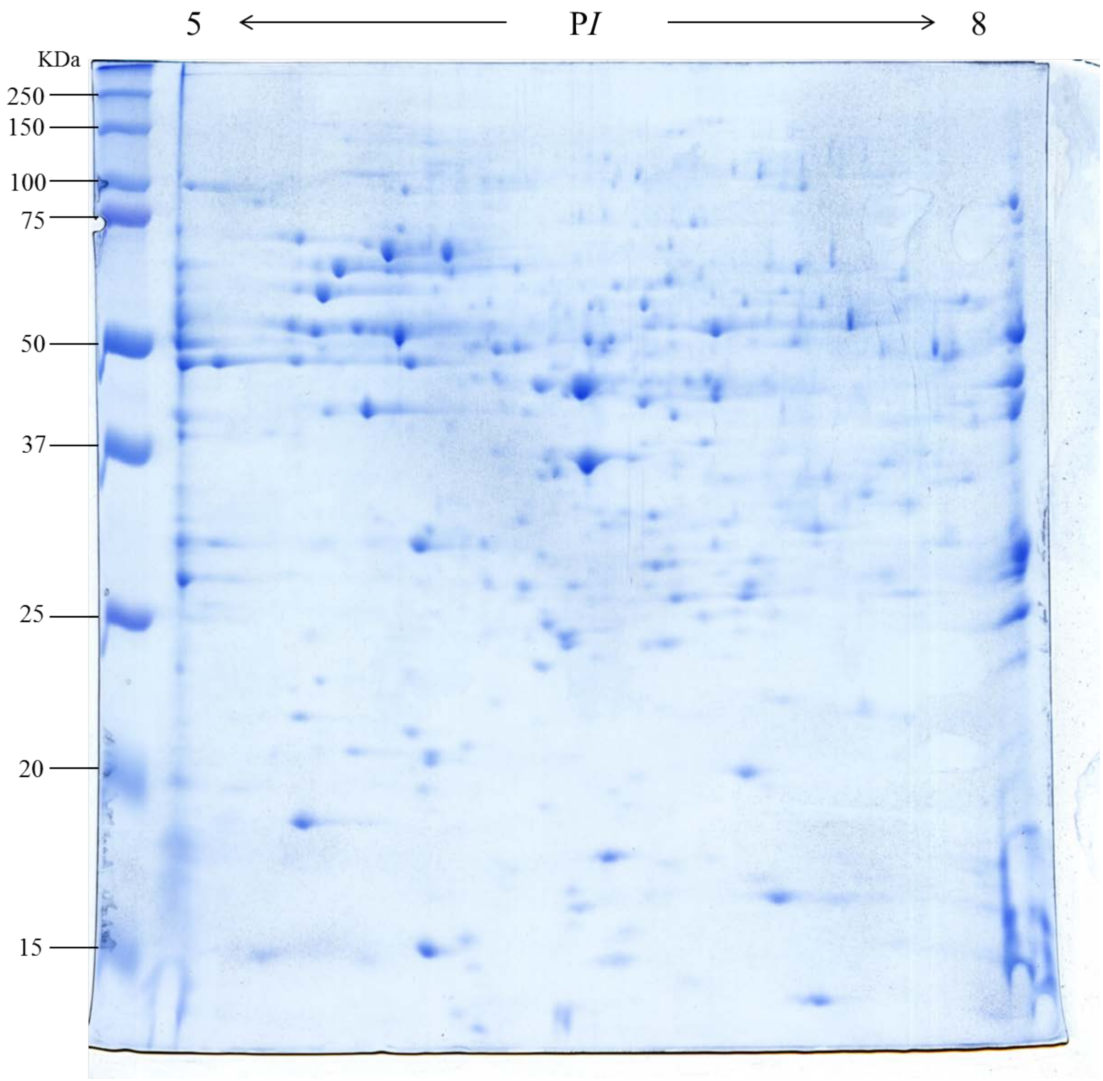

P50+:

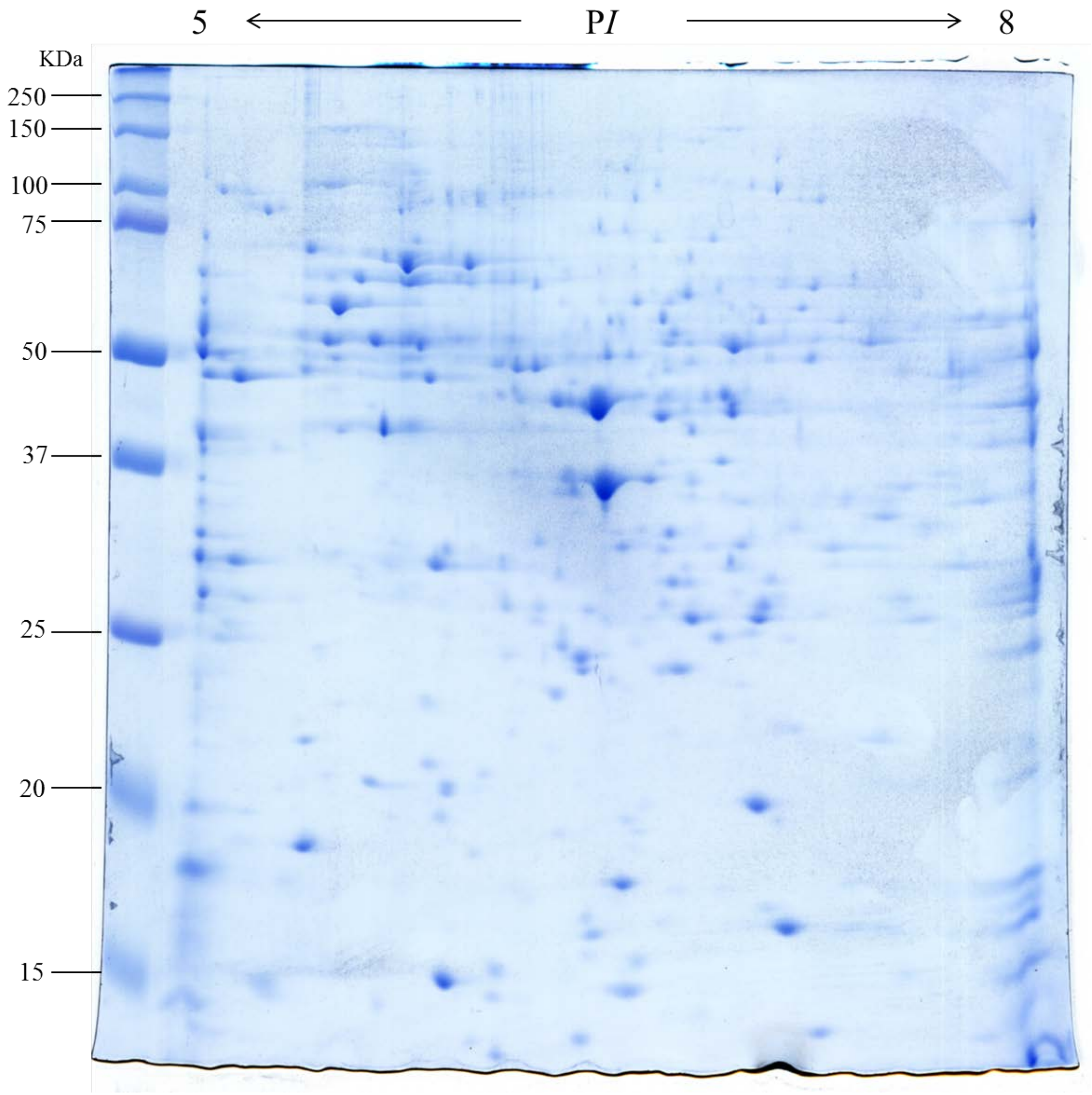

BC9-:

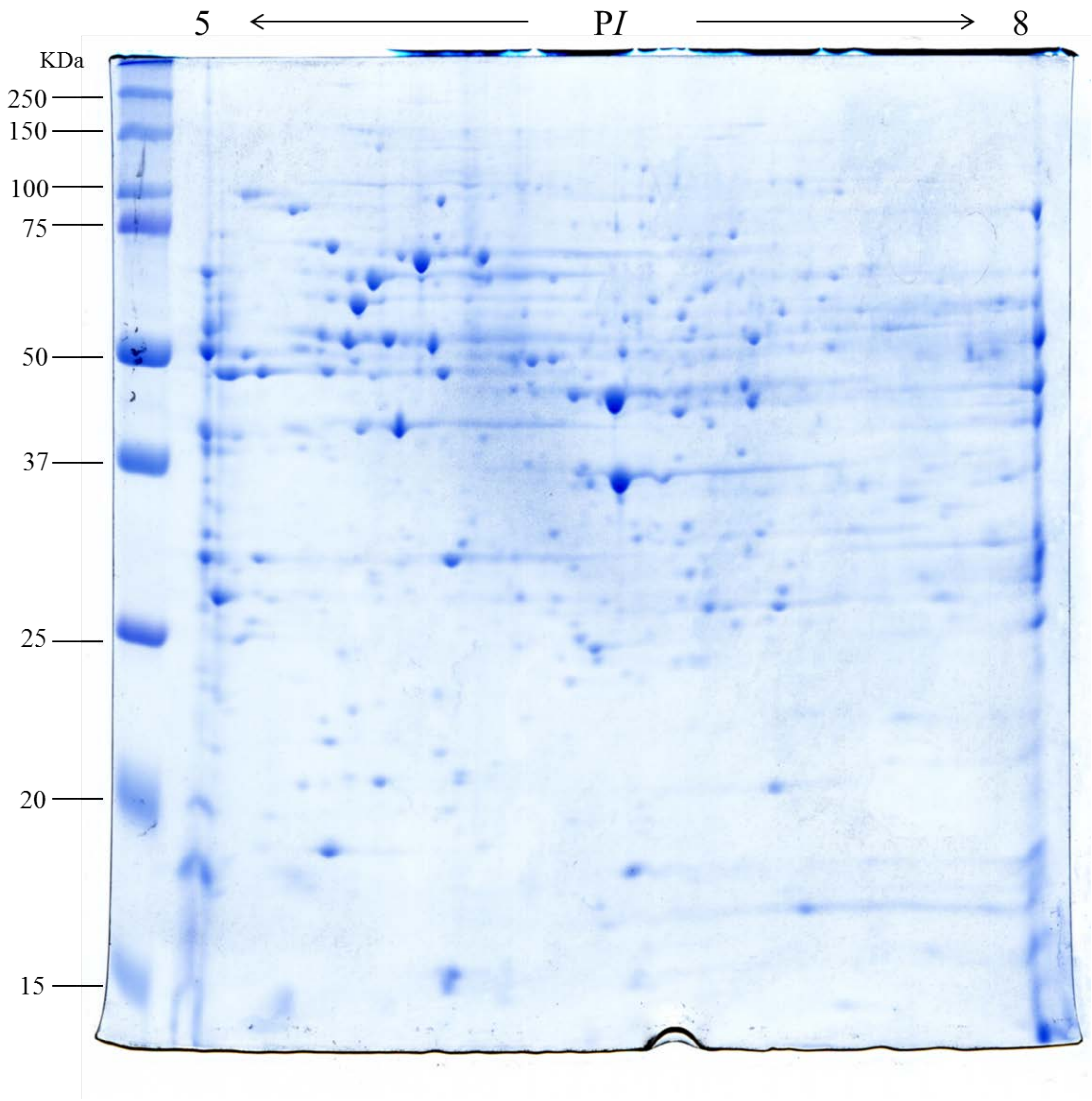

BC9+:

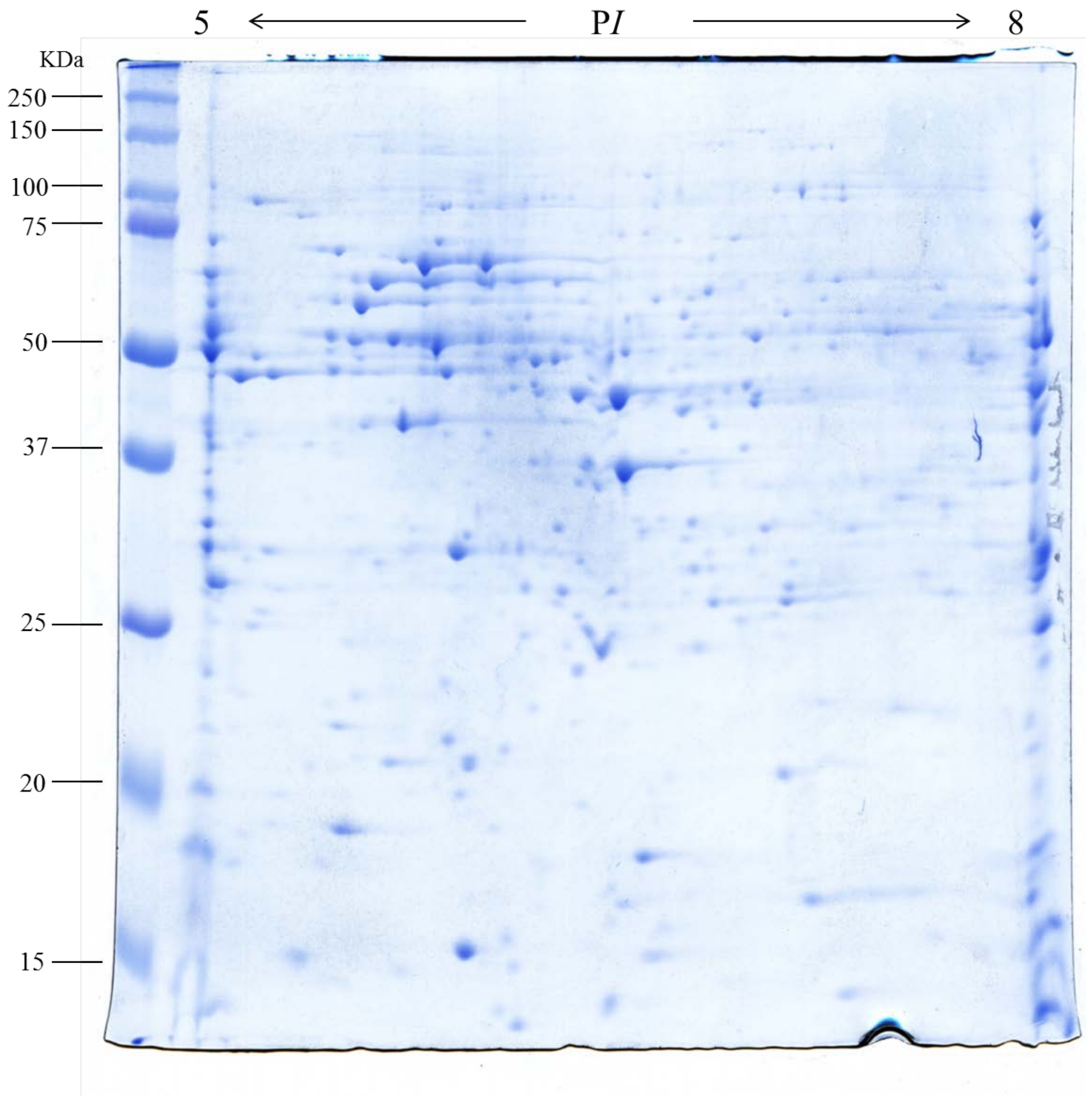

Figure S2. The full-length gels of mitochondria in P50 and BC9 following BmNPV infection.

P50-:

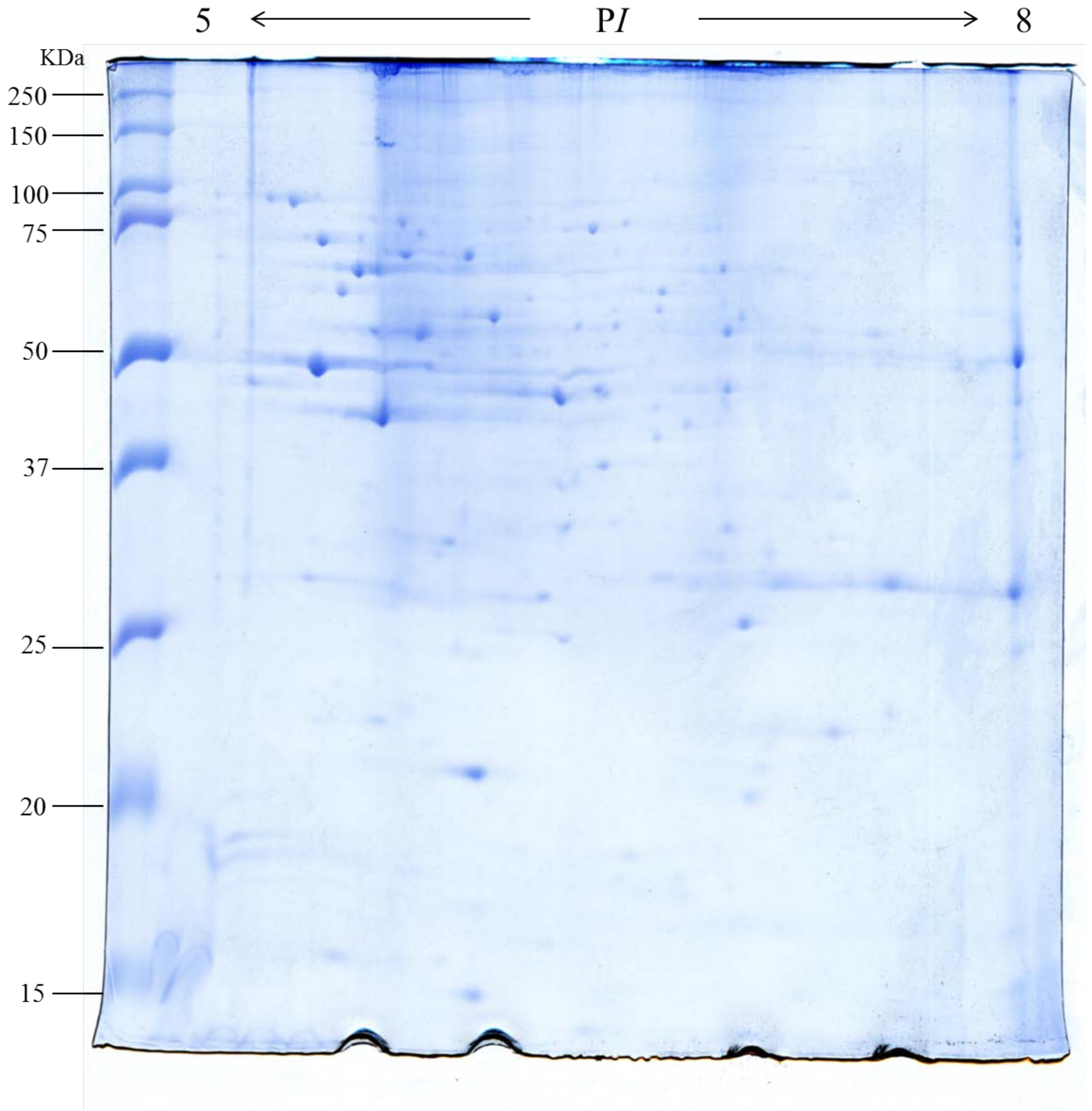

P50+:

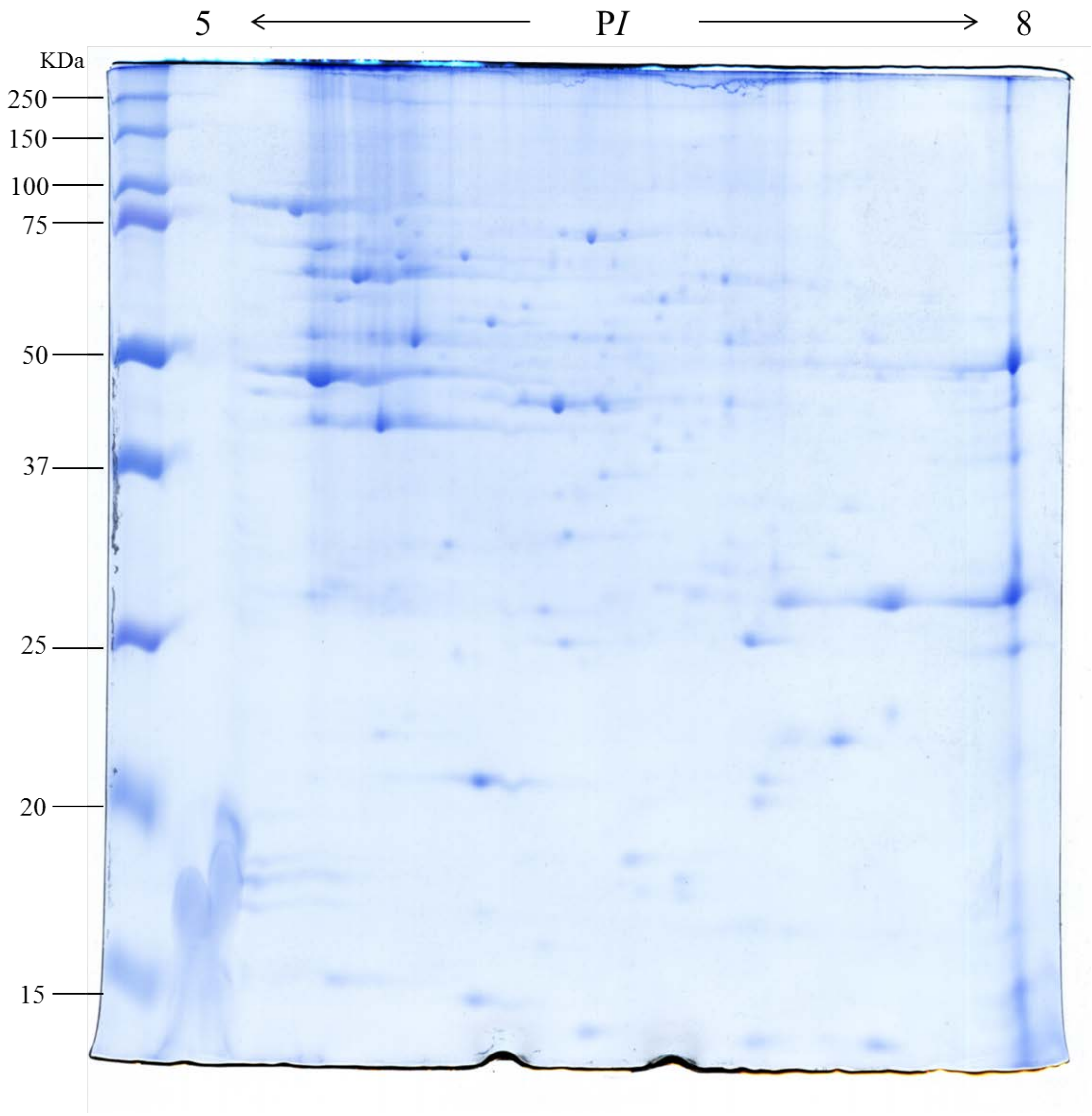

BC9-:

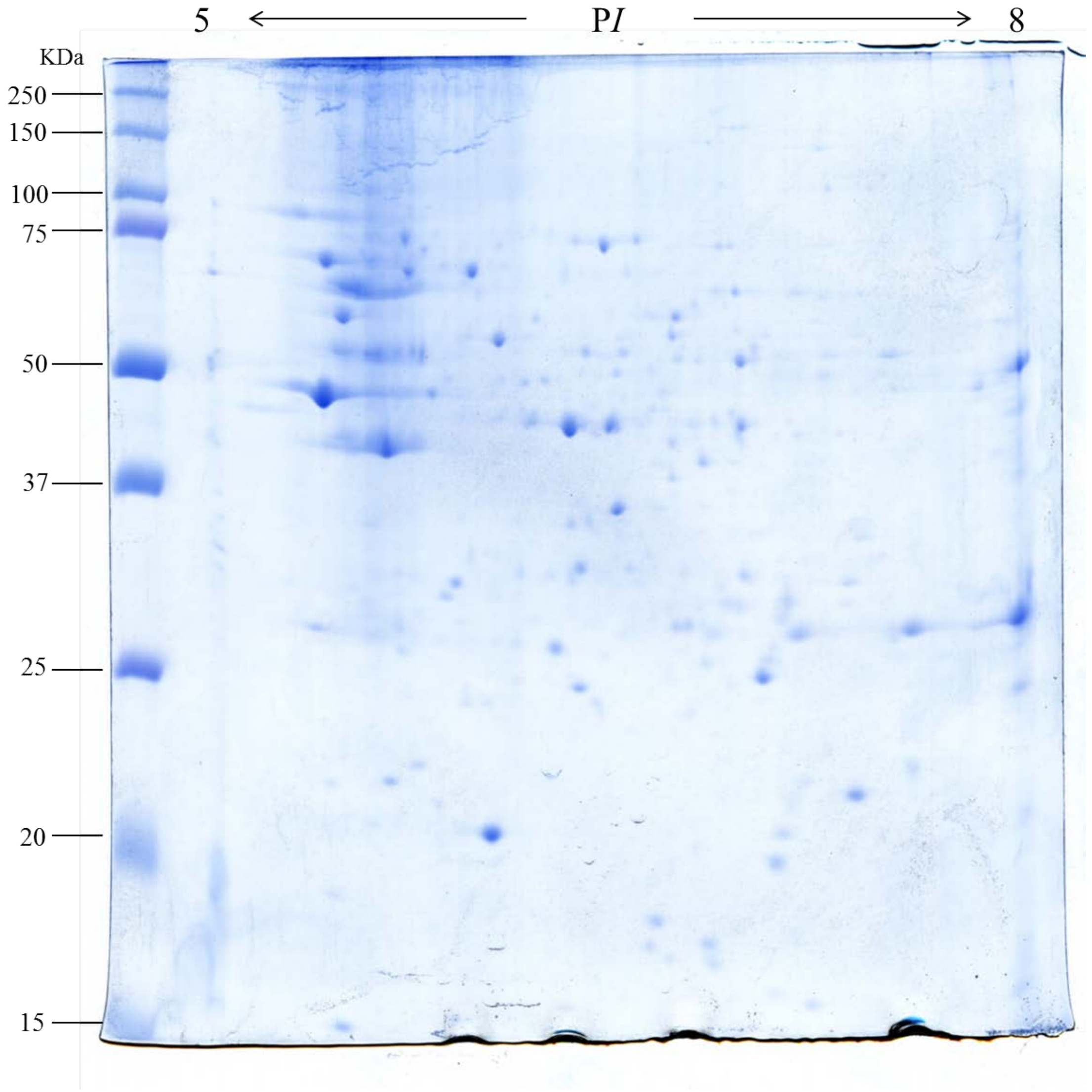

BC9+:

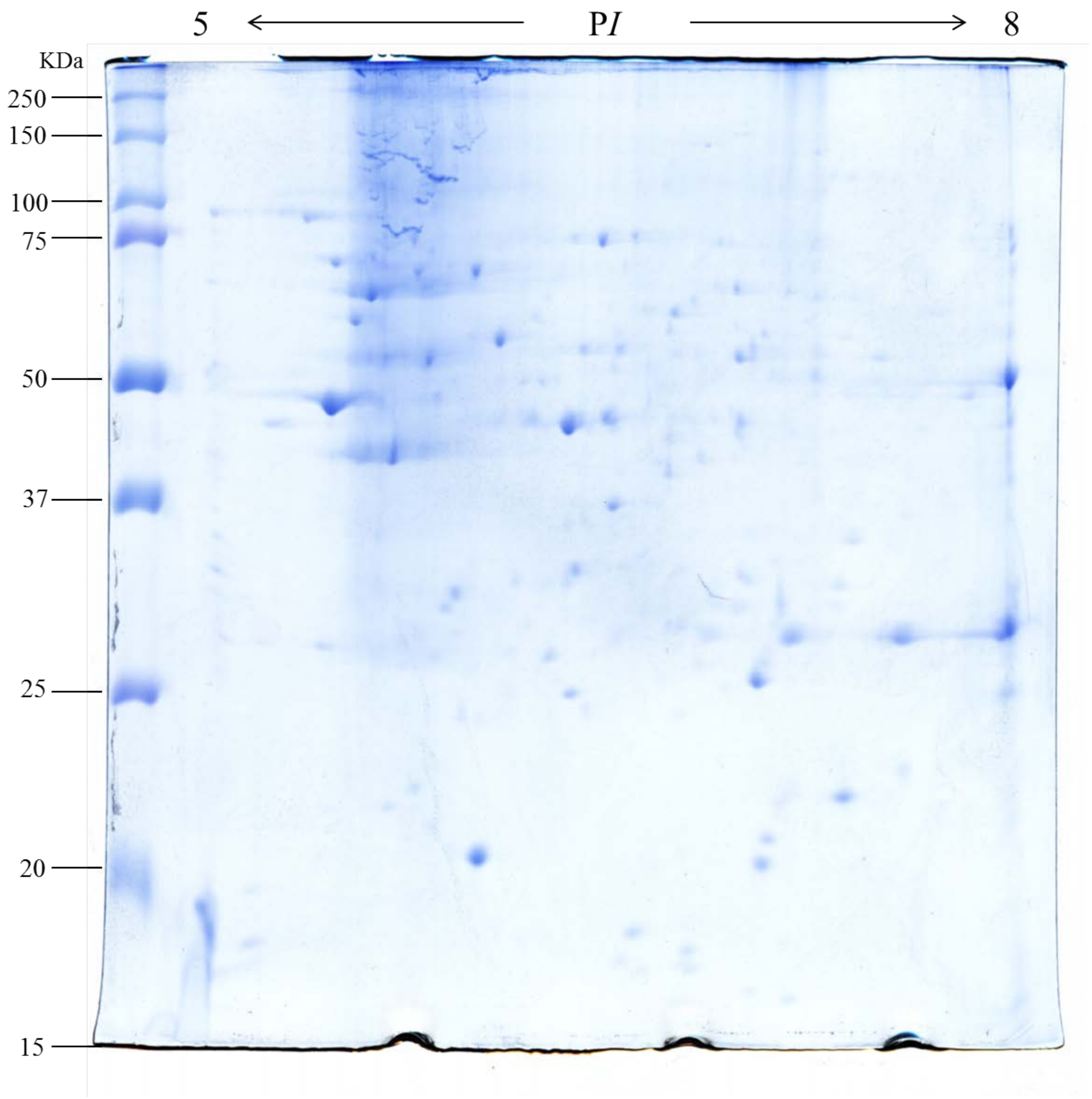

Figure S3. The full-length gels of microsome in P50 and BC9 following BmNPV infection.

P50-:

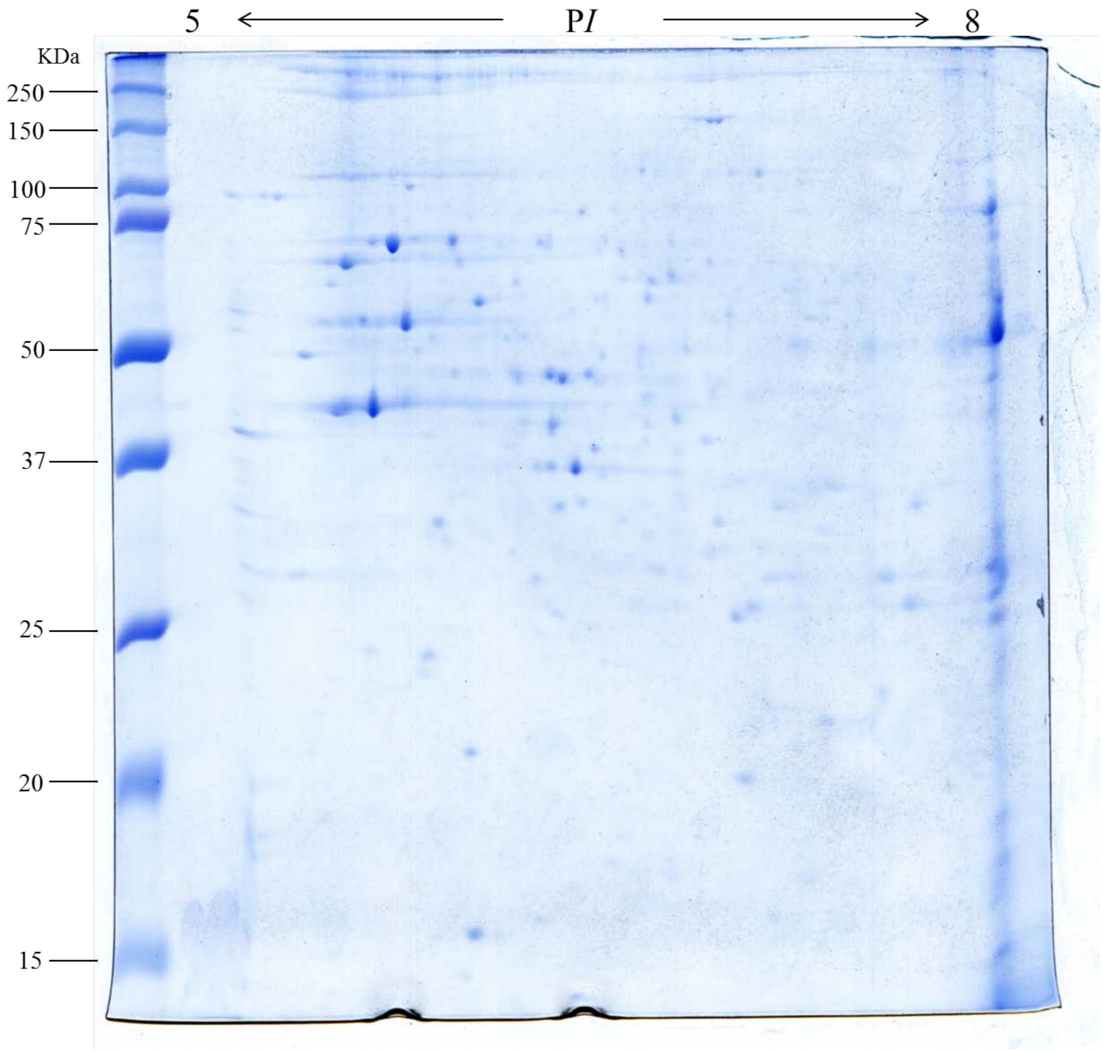

P50+:

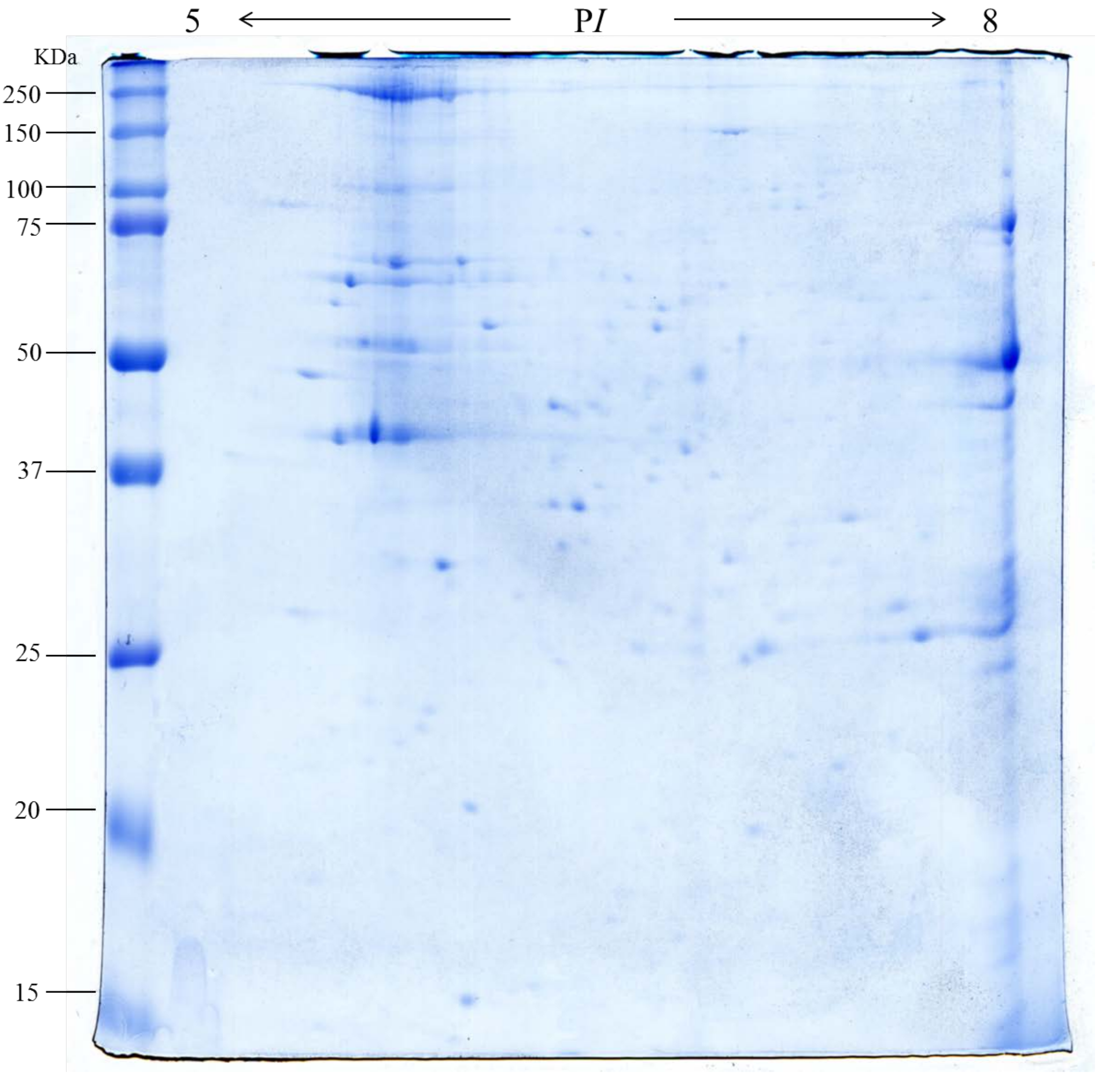

BC9-:

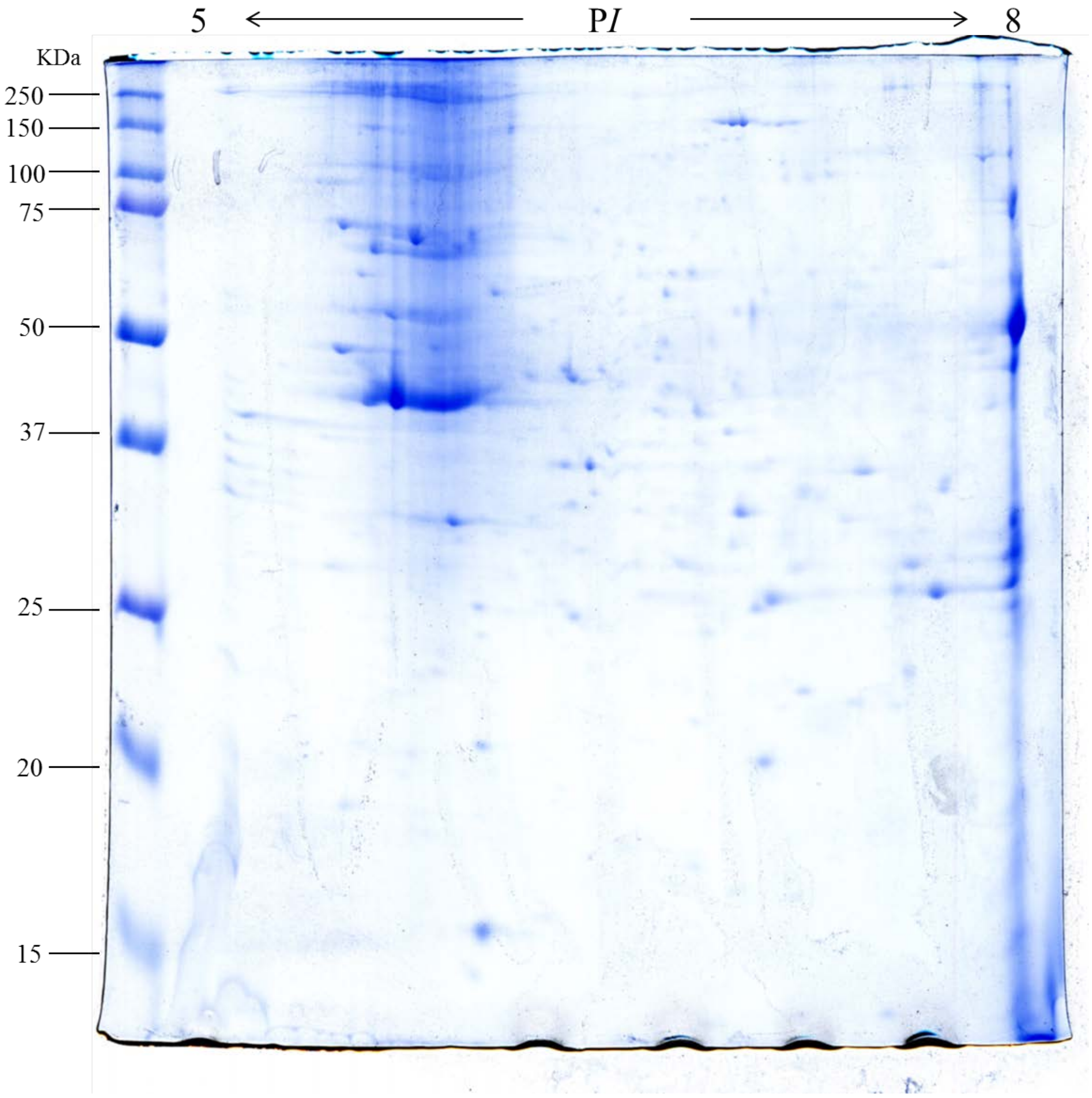

BC9+:

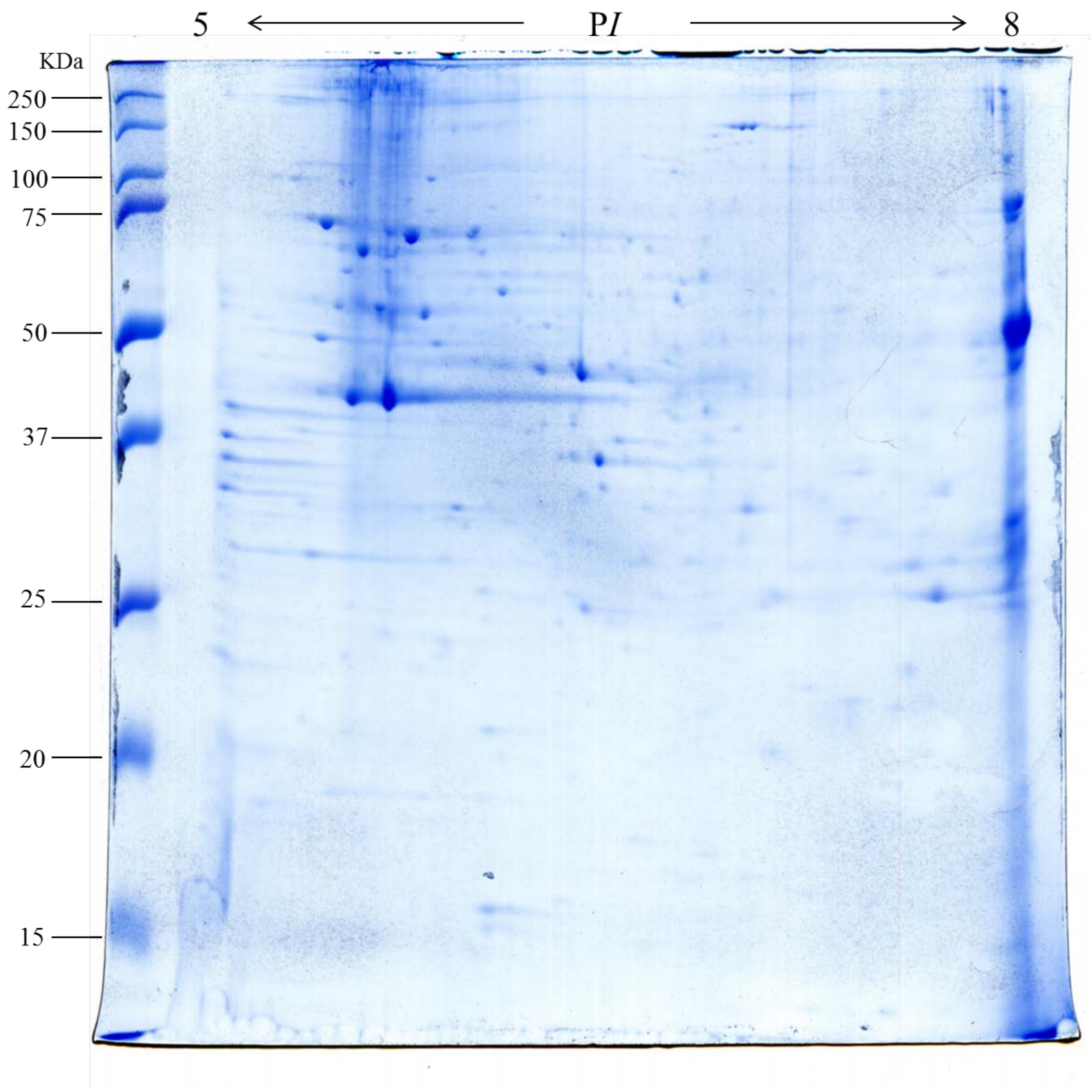

Supplement: Supplementary Tables [file srep45690-s1.pdf]
